# Integration of Fuzzy Matter-Element Method and 3D-QSAR Model for Generation of Environmentally Friendly Quinolone Derivatives

**DOI:** 10.3390/ijerph17093239

**Published:** 2020-05-06

**Authors:** Xixi Li, Baiyu Zhang, Wendy Huang, Cuirin Cantwell, Bing Chen

**Affiliations:** Northern Region Persistent Organic Pollution Control (NRPOP) Laboratory, Civil Engineering, Faculty of Engineering and Applied Science, Memorial University, St. John’s, NL A1B 3X5, Canada; xl7724@mun.ca (X.L.); wendyh@mun.ca (W.H.); cdcantwell@mun.ca (C.C.); bchen@mun.ca (B.C.)

**Keywords:** quinolone antibiotics, infrared characteristic vibration spectrum, ultraviolet absorption spectrum, fuzzy matter-element method, three-dimensional quantitative structure–activity relationship, molecular modification

## Abstract

The environmental pollution of quinolone antibiotics (QAs) has caused rising public concern due to their widespread usage. In this study, Gaussian 09 software was used to obtain the infrared spectral intensity (IRI) and ultraviolet spectral intensity (UVI) of 24 QAs based on the Density Functional Theory (DFT). Rather than using two single-factor inputs, a fuzzy matter-element method was selected to calculate the combined effects of infrared and ultraviolet spectra (CI). The Comparative Molecular Field Analysis (CoMFA) was then used to construct a three-dimensional quantitative structure–activity relationship (3D-QSAR) with QAs’ molecular structure as the independent variable and CI as the dependent variable. Using marbofloxacin and levofloxacin as target molecules, the molecular design of 87 QA derivatives was carried out. The developed models were further used to determine the stability, functionality (genetic toxicity), and the environmental effects (bioaccumulation, biodegradability) of these designed QA derivatives. Results indicated that all QA derivatives are stable in the environment with their IRI, UVI, and CI enhanced. Meanwhile, the genetic toxicity of the 87 QA derivatives increased by varying degrees (0.24%–29.01%), among which the bioaccumulation and biodegradability of 43 QA derivatives were within the acceptable range. Through integration of fuzzy matter-element method and 3D-QSAR, this study advanced the QAs research with the enhanced CI and helped to generate the proposed environmentally friendly quinolone derivatives so as to aid the management of this class of antibiotics.

## 1. Introduction

Quinolone antibiotics (QAs) are a new class of artificially synthesized anti-infective drugs. They are widely used in the treatment of various infectious diseases due to their broad bactericidal spectrum, strong antibacterial properties, low toxicity, few side effects, and low price [1]. They are a class of antibiotics widely applied in veterinary medicine. After usage, QAs will remain in the animal as their original molecular structures or pass through feces and urine in the form of metabolites. These QAs and their metabolites will excrete into the external environment [2]. According to Xander et al. [3], 47 research papers have reported the distribution of QAs in the environment and found that their residues are concentrated worldwide including in Pearl River, Huangpu River, Xiaoqing River, Hai River, Baiyangdian Lake, and Dongting Lake in China; densely populated watersheds such as Lake Ontario in North America, Taft River and in Europe, and Mekong River in Asia. Zhang et al. [4] used the III fugacity model to study the distribution characteristics of QAs in 58 watersheds in China. The results showed that after metabolism in humans and animals, the total amount of QAs excreted from feces and urine is 5.4 × 10^7^ kg. Only 0.02 × 10^7^ kg are removed in wastewater treatment, while the remaining 5.38 × 10^7^ kg still directly enter the receiving environment. Chen et al. [5] used gas chromatography and mass spectrometry to detect QA concentrations in 19 key groundwater monitoring wells in Beijing. The average concentrations of the QAs ciprofloxacin and norfloxacin were found to be 4.9 and 0.2 ng, respectively. In addition, studies have shown that the levels of norfloxacin, ciprofloxacin, and enoxacin in groundwater in Spain, were 64.13, 38.93, 44.47 ng, respectively [6]. Norfloxacin, ciprofloxacin, and enoxacin in the groundwater of a Swedish pharmaceutical company reached 31, 14,000, and 1900 ng, respectively [7]. These studies have shown that the high residual concentrations of QAs are a threat to the environment. QAs accumulate in various aquatic organisms, however, the concentration of different QAs are quite different. QAs, including ofloxacin (2.72 µg/kg), enrofloxacin (762.34 µg/kg), and ciprofloxacin (3.08 µg/kg) were found in bivalve samples [8]. A mean value of ofloxacin (5.58 µg/kg), enrofloxacin (3.08 µg/kg), ciprofloxacin (4.17 µg/kg), and norfloxacin (23.8 µg/kg) were found in the muscles of fish, turtles, and birds from Baiyangdian Lake, China [9]. Li et al. [10] found that among 22 antibiotics, QAs had the highest concentration in 190 mollusks samples with a mean concentration of 86.76 μg/kg dry weight. The investigation of antibiotics in mollusks from Bohai Sea, China, indicated that the mean concentrations of QAs in the mollusks were in the order of “NOR > OFL > CIP > FLE > SAR > LOM > ENR > DIF”. The bioaccumulation capacity of QAs is highly relevant to their molecular structures [10]. Therefore, it is of critical importance that the relationship between QAs molecular structures and their characteristics can be identified, so that environmentally friendly QA derivatives can be generated.

3D quantitative structure–activity relationship (3D-QSAR) models have been utilized to design and develop potent drugs by correlating 3D-structural features of the chemicals with properties of interest [11]. Among the existing 3D-QSAR methods, the Comparative Molecular Field Analysis (CoMFA) and the Comparative Molecular Similarity Indices Analysis (CoMSIA) are extensively used in the current practice of rational drug design [12]. 3D-QSAR has been applied to design environmentally friendly molecules. Wang et al. [13] designed a pentachlorophenol molecule with lower bioaccumulation by 3D-QSAR. Tong et al. [14] studied the design of HIV protease inhibitors by using a certain 3D-QSAR method (CoMFA or CoMSIA). Gu et al. [15] predicted the octanol-water partition coefficient for polychlorinated naphthalenes through 3D-QSAR models. However, there are still very limited studies about the use of 3D-QSAR to predict the environmentally friendly properties of QAs. Zhao et al. [16] combined QSAR/QSPR with molecular docking to examine the biodegradability of C20-carbonyl and the C21-carboxyl groups of fluoroquinolones. Zhao et al. [17] designed new fluoroquinolones using SYBYL-X 2.0. After changing the molecular groups, the photodegradability of new fluoroquinolones increased from 15.04% to 40.92% [17].

To construct a 3D-QSAR model for QA analysis, effective methods for detection of QAs in environmental samples are needed so as to obtain infrared and ultraviolet spectral characteristics of QAs to build the database. Infrared and ultraviolet spectral based analytical methods have been well developed for QA detection. Claine [18], Efthimiadou et al. [19], Skyrianou et al. [20], and Zampakou et al. [21] conducted infrared spectroscopy analysis to detect QAs. Bailac et al. [22] detected quinolones in chicken tissues by liquid chromatography based on ultraviolet spectral analysis. Zampakou et al. [21] studied the interaction of quinolone antimicrobial complexes with calf-thymus deoxyribonucleic acid investigated by ultraviolet spectroscopy. However, 3D-QSAR model development needs a large amount of infrared and ultraviolet spectral characteristics of QAs as inputs. Obtaining such information is expensive and time-consuming. Rather than using experimental analysis to generate infrared and ultraviolet spectral data, theoretical calculation methods [23,24] can assist QAs quantification to speed up the database generation. Among them, the Density Functional Theory (DFT) based calculation has been widely adopted. Qiu [25] et al. used DFT to calculate the infrared and ultraviolet spectra of phthalate esters and their derivatives, and used 3D-QSAR to analyze the spectral changes of phthalates before and after substitution. Yang [26] et al. used DFT to calculate the infrared spectra of 36 polybrominated biphenyl molecules and recorded the highest infrared vibration intensity of each molecule.

DFT can help to obtain either infrared or ultraviolet spectra of QAs. These infrared or ultraviolet spectra information can then feed a 3D-QSAR model as a single-factor input. However, instead of using two single-factor inputs (i.e., infrared spectra intensities and ultraviolet spectra intensities), an infrared-ultraviolet integrated-factor is desired to increase the efficiency and accuracy of a 3D-QSAR model. It is thus essential to introduce an effective method for integrating infrared and ultraviolet spectral characteristics accurately. The fuzzy matter-element analysis theory can serve this purpose. It can solve the multiple parameter evaluation problem of incompatibility through establishing the corresponding matter-element [27]. Yang et al. [28] used the fuzzy matter-element method to assess the water resources carrying capacity of six regions in Huai River, China. Liu and Zou [29] used the fuzzy matter-element evaluation method to assess water quality, obtaining very similar results to the official reported water quality. Compared with the traditional method for superscale calculation, the fuzzy matter-element analysis method has lower workload and would overcome the adverse effects from abnormal values. The integration of fuzzy matter-element method and 3D-QSAR models has a great potential for generating more accurate results, though the topic has not been tackled in previous studies.

In this study, 24 QAs were selected to build the 3D-QSAR models, and among them, marbofloxacin and levofloxacin were selected as the target molecules to be modified. DFT was used obtain infrared and ultraviolet spectra intensities of 24 QAs. The fuzzy matter-element method was adopted to deal with the magnitude of single spectrum (infrared spectral intensity (IRI) and ultraviolet spectral intensity (UVI)) intensities, and the combined effect (CI) values that were obtained. In addition, a 3D-QSAR model based on CI was constructed, and the substituted sites of QAs (improving the CI values) were precisely obtained based on the contour map. The associated CI, IRI, and UVI were investigated and compared. Eventually, the stability, functionality (genetic toxicity), and environmental friendliness (bioaccumulation and biodegradability) of all QA derivatives were evaluated in order to generate more environmentally friendly QA derivatives. This study considers both functionality and environmental friendliness of QA derivatives which leads to a tool for a generation of new antibiotics in the future.

## 2. Materials and Methods

### 2.1. Data Sources

The theoretical calculations of the infrared and ultraviolet spectra of the 24 QAs studied in this paper are based on Gaussian-09 software (Gaussian Inc., Wallingford, CT, USA). Gaussian is the most widely used software package for computational and quantum chemistry [30]. Gaussian can be used to process larger molecular weight structures such as anthraquinone, polyhydroxylated anthroquinones, and polyfluorinated dibenzo-p-dioxins for research and calculation [24,31]. DFT is a classical computational method of electronic structure theory which has been frequently used [31]. In most DFT calculations today, the combination of Gaussian orbits is commonly used to represent atomic orbits [32]. The infrared spectrum and ultraviolet spectrum are optimized for the structure of QAs in a gaseous environment at the B3LYP/6-311G (d) level. The infrared spectrum is the optimal structure and its value is calculated and corrected by a correction factor of 0.9614. The ultraviolet spectrum is based on the optimized ground-state and excited-state geometry, calculated using the DFT method [32].

### 2.2. Calculation of the CI Based on Fuzzy Matter-Element Method

#### 2.2.1. Construction of Composite Fuzzy Matter-Element Matrix about QAs’ CI

The elementary elements of an ordered triad R = (*M*, *C*, *q*) are described by the three matter-elements: quinolones (*M*), detection type (*C*), and spectral intensity (*q*) [33]. There are two types of detection methods of quinolone *M*: infrared spectrum (*C*_1_) and ultraviolet spectrum (*C*_2_), corresponding spectral intensities *q*_1_*, q*_2_*, …, q*_24_, and *R* is called m-dimensional fuzzy matter-element. When all the quantities in the composite matter-element (*R_m_*) have ambiguity, then *R_m_* is called a composite fuzzy matter-element and can be recorded as:

(1) $$R_{mn}=\left[\begin{array}{ccc} \;M_{1}\; & M_{2}\;\cdots & M_{24}\\ C_{1}\;q_{11} & q_{12}\;\cdots & q_{124}\\ C_{2\;}q_{21} & q_{22}\;\cdots & q_{224} \end{array}\right]$$

where *R_mn_* is m dimensional matter-element for 24 QAs, *C_j_* is the detection type of *j*-th characteristic (*j* = 1, 2), *M_i_* the *i*-th molecule (*i* = 1, 2, …, 24), *q_ij_* is the *i*-th spectral intensity corresponding to the *j*-th detection type of a molecule.

#### 2.2.2. Determination of Subordination Membership Degree of QAs’ CI

The subordination membership degree indicates the fuzzy value corresponding to the single effect parameter of the infrared and ultraviolet spectrums belonging to the standard value of each single effect parameter. The degree of membership is represented by *η_ij_*. The fuzzy matter-element matrix *R’_mn_* of the preferential membership can be written as:

(2) $$R^{\prime }{}_{mn}=\left[\begin{array}{ccc} \;M_{1}\; & M_{2}\;\cdots & M_{24}\\ C_{1}\;\eta_{11} & \eta_{12}\;\cdots & \eta_{124}\\ C_{2\;}\eta_{21} & \eta_{22}\;\cdots & \eta_{224} \end{array}\right]$$

where *R’_mn_* is the priority membership fuzzy matter-element; *η_ij_* is the priority membership of the *j*-th detection type of the *i*-th molecule belonging to the standard sample, *i* = 1, 2, …, 24; *j* = 1, 2. The priority membership can be recorded as:

(3) $$\{\begin{array}{c} \eta_{ij}=\frac{q_{ij}}{\mathrm{Max}q_{ij}}\;(\mathrm{higher}\;\mathrm{is}\;\mathrm{the}\;\mathrm{better})\\ \eta_{ij}=\frac{\mathrm{Min}q_{ij}}{q_{ij}}\;(\mathrm{lower}\;\mathrm{is}\;\mathrm{the}\;\mathrm{better}) \end{array}$$

where *Maxq_ij_* represents the maximum value of the spectral intensity in each detection type corresponding to each molecule; *Minq_ij_* represents the minimum value of the spectral intensity in each detection type corresponding to each molecule. It is easier to detect the effect of infrared and ultraviolet spectra, when the value of the effects is larger. Therefore, the largest index will be used for further calculation.

#### 2.2.3. Determination of Standard Fuzzy Matter-Element and Variance Compound Fuzzy Matter-Element Matrix for the CI of QAs’ IRI and UVI

The m-dimensional standard fuzzy matter-element *R*_0*m*_ is the maximum or minimum value of the spectral strength of the superiority of the spectral intensity in each detection type in the fuzzy matter-of-priority membership. The form is as follows:

(4) $$R_{0m}=\left[\begin{array}{c} \;M_{0}\\ \;C_{1}\;\eta_{01}\\ C_{2}\;\eta_{02} \end{array}\right]$$

where *M*_0_ represents the standard sample; *η*_0*j*_ represents the maximum value of the membership degree of the *j*-th detection type.

*R_Δ_* of variance compound fuzzy matter element can be written as:

(5) $$R_{\Delta }=\left[\begin{array}{ccc} \;M_{1}\; & M_{2}\;\cdots & M_{24}\\ C_{1}\;\Delta_{11} & \Delta_{12}\;\cdots & \Delta_{124}\\ C_{2\;}\Delta_{21} & \Delta_{22}\;\cdots & \Delta_{224} \end{array}\right]$$

where *Δ_i__j_*= (*η*_0*j*_-*η_ij_*)^2^; because the largest subordination membership degree of this paper is the most optimum, *η*_0*j*_ equals to 1.

#### 2.2.4. Determination of Weighting for Evaluation Index of the CI of QAs’ IRI and UVI

The weight determination methods include a subjective weighting approach and objective weighting approach [34]. The subjective weighting approach in this paper uses the empirical method and the objective weighting approach uses the entropy method. The entropy method is calculated according to the following steps:

(1) Construct a judgment matrix *R* for the infrared spectrum and ultraviolet spectrum of 24 QAs, and normalize *R* to obtain a normalized judgment matrix A. Determine the entropy of the *j*-th detection type according to the definition of entropy:

(6) $$S_{j}=\frac{1}{lnn}\left\{\sum_{i=1}^{n}\left[\frac{1+a_{ij}}{\sum_{i=1}^{n}\left(1+a_{ij}\right)}ln\frac{1+a_{ij}}{\sum_{i=1}^{n}\left(1+a_{ij}\right)}\right]\right\}\;\left(i=1,\;2,\;\ldots ,\;24;\;j=1,2\right)$$

where *S_j_* is the entropy of the *j*-th detection type; *a_ij_* is the fuzzy value of each item in the normalized judgment matrix.

(2) Calculate the entropy weighting of each detection type, it can be recorded as follows:

(7) $$w_{j}=\frac{1-S_{j}}{m-\sum_{j=1}^{m}S_{j}}\;\left(0\leq w_{j}\leq 1,\;\sum_{j=1}^{m}w_{j}=1\right)$$

where *w_j_* is the entropy of the *j*-th detection type, *j* = 1, 2.

(3) Determine the comprehensive weights of the comprehensive effect parameters of QAs’ infrared and ultraviolet spectra:

The objective weighting *w_j_* determined by the entropy method is combined with the subjective weighting *θ_j_* determined by the empirical method. The comprehensive weighting *w’_j_* of each detection type is finally determined as:

(8) $$w^{\prime }{}_{j}=\xi \theta_{j}+\left(1-\xi \right)\;w_{j}\;\left(j=1,2\right)$$

where ξ is the preference coefficient of the subjective weighting ξ∈[0,1]. A larger ξ value indicates that the research focuses more on subjective weighting; conversely, a lower ξ indicates that decision makers should pay more attention to objective weighting.

#### 2.2.5. Euclidean Closeness Calculation for the CI of QAs’ IRI and UVI

The Euclidean closeness was used to comprehensively analyze the infrared and ultraviolet spectra of 24 QAs. The calculation formula of Euclidean closeness *e_i_* can be determined as follows:

(9) $$e_{i}=1-\sqrt{\sum_{j=1}^{m}w^{\prime }{}_{j}\Delta_{ij}}\;\left(i=1,\;2,\;\ldots ,24;\;j=1,2\right)$$

### 2.3. Construction of 3D-QSAR Model to Generate Environmentally Friendly QAs

Based on the database generated from Section 2.2, a 3D-QSAR model was constructed in this paper. The 3D-QSAR analysis was performed using SYBYL-X2.0 software (Tripos Company, St. Louis, USA) [35]. The comprehensive effects of infrared and ultraviolet spectra of 24 QAs were calculated based on the fuzzy matter-element method. When calculating the parameters of the CoMFA field, partial least squares (PLS) analysis was used to establish the relationship between the structure of the target compound and the biological activity [36]. When using PLS analysis, the Leave-One-Out method was used to cross-validate the training set compounds and calculate the cross-validation coefficient *q^2^* and the number of optimal principal components *n* [37]. Non-cross-validation regression (No Validation) was then used to perform regression analysis and calculate the non-cross-validation coefficient *r^2^*, standard deviation *SEE*, and test value *F*, in order to complete the establishment of the CoMFA model [38]. In addition, single-effect models of infrared and ultraviolet spectra also follow the above operations to construct CoMFA models of single-effects of infrared and ultraviolet spectra of QAs.

## 3. Results and Discussion

### 3.1. Calculation of QAs’ IRI and UVI based on DFT

There are many types of QAs, many of which have different behavioral characteristics and hazards in the environment. Therefore, determining the types of QAs that exist in the environment and extracting spectral information assists prediction and evaluation of the magnitude of the hazards caused by such substances to the environment. In this study, the UVI of 24 QAs were obtained using the DFT method based on the optimized ground-state and excited-state geometry. The IRI of 24 QAs were calculated using the Gaussian 09 software with a correction factor of 0.9614. The intensities of both the infrared and ultraviolet spectrum were optimized in a gaseous environment at the B3LYP/6-311G (d) level. Table 1 lists the calculation results of infrared and ultraviolet spectral intensities of 24 QAs.

### 3.2. Determination of CI of QAs’ IRI and UVI Based on Fuzzy Matter-Element Method

#### 3.2.1. Subordination Membership Degree, Standard Fuzzy Matter-Element and Variance Compound Fuzzy Matter-Element Matrix

Two characteristics of QAs, infrared and ultraviolet spectrums, were applied and the associated fuzzy membership degree, standard fuzzy matter-element and variance composite fuzzy matter-element matrix of the two spectral intensities were calculated using the fuzzy matter-element method according to Equations (1)–(9). The calculation results are summarized in Table 2, which were used to further calculate the CI of QAs.

#### 3.2.2. Comprehensive Weightings for CI of QAs’ IRI and UVI

The data of the infrared and ultraviolet spectra of QAs are derived from the results of DFT calculations in quantum chemical calculations. Both detection methods have shown similar physical significance when detecting quinolones [39,40]. Therefore, the subjective weighting for both infrared and ultraviolet spectra was set as 0.5. In addition, the obtained data was normalized before calculating the objective weightings. The specific results are shown in Table 3.

#### 3.2.3. CI of QAs’ IRI and UVI

In this study, the Euclidean closeness was calculated by using Equation (9) with the processed single-effect parameters of 24 QAs representing the comprehensive effects of infrared and ultraviolet spectra of these QAs. As presented in Table 4, the CI values obtained based on the fuzzy matter-element method varied from 0.1613 to 0.8958. The dynamic range of the CI values is 5.6, greater than that stated in previous studies [41,42,43,44,45]. It indicated that the CI data set of QAs’ IRI and UVI could be employed to build the associated 3D-QSAR model based on the comprehensive effect.

### 3.3. Construction and Evaluation of 3D-QSAR Model Based on CI of QAs’ IRI and UVI

#### 3.3.1. Construction of the 3D-QSAR Model

**Figure 1:**
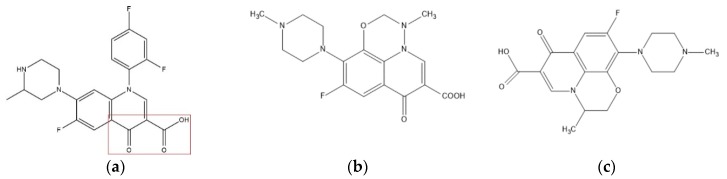
(**a**) Molecular structure and common skeleton of Temafloxacin; (**b**) molecular structure of marbofloxacin; (**c**) molecular structure of levofloxacin.

The CI of 16 QAs were randomly selected as data sources. Temafloxacin with the highest CI (*e*_24_ = 0.7296) was chosen as the target molecule, 13 QAs as the training set, and the remaining 4 QAs as the testing set (Temafloxacin exists in both the training set and the testing set). Based on these comprehensive effects, a 3D-QSAR model was constructed. SYBYL-X2.0 (Tripos Company, USA) was used in this study to select the lowest energy conformation of the molecule as the dominant stable conformation, and G-H electrical charges (Gasteiger–Huckel) were loaded. The maximum number of optimizations was 10,000 using the Powell energy gradient method, and the energy convergence was limited to 0.005 kJ/mol [46,47]. The optimized molecules were stored in the database for alignment. All molecules were aligned with the pharmacophore characteristic elements in the marked area shown in Figure 1 as the basic skeleton. The structures of selected target molecules, marbofloxacin and levofloxacin are also shown in Figure 1. The CoMFA module will be used to establish the infrared and ultraviolet spectra suitable for QAs by using the basic skeleton for comprehensive effects prediction.

#### 3.3.2. Performance Evaluation of the 3D-QSAR Model

The evaluation results of 3D-QSAR model show that in the CoMFA model, the cross-validation coefficient *q^2^* is 0.67 (*q^2^* > 0.5), and the best principal component *n* is 3, indicating that the model has a good prediction ability. The cross-validation coefficient *R^2^* is 0.984 (>0.9), the standard error of estimate (*SEE*) is 0.023, and the F-test value is 179.271, indicating that the model has a good ability to fit and predict [48].

Golbraikh and Ropsha [49] confirmed that the strict QSAR model verification procedures should include internal and external verification. The use of internal verification parameters such as *q^2^* cannot assess the quality of the model. Therefore, external verification methods need to be applied. External verification methods are one of the most valuable verification methods, and were applied to evaluate the predictive ability of the obtained model. The overall predictive ability of the CoMFA model was externally verified by predicting the activity of the compounds in the independent testing set [49]. The predictive ability of the model is represented by the predicted *r^2^_pred_*, and its calculation formula is as follows:

(10) $$r_{pred}^{2}=1-\frac{PRESS}{SD}$$

In the formula, *PRESS* refers to the sum of the squared deviations between the calculated and predicted values in the test set, and *SD* refers to the sum of the squared deviations between the calculated values of the compounds in the test set and the average values of the calculated values of the compounds in the training set.

The constructed CoMFA model was used to predict the activity of the test set molecules, and external verification was performed based on the prediction results (Table 5). The results show that the interaction test coefficient *r^2^_pred_* of the external prediction set is 0.9695 (>0.6) [50]. Tropsha method was also used to evaluate the external prediction ability of this model. The calculation results indicated that r was 0.9932, r^2^ was 0.9864 (>0.6), k was 1.0269 (0.85 < k < 1.15), k’ was 0.9712 (0.85 < k’ < 1.15), r_0_^2^ was 0.893, and r_0′_^2^ was 0.932. Furthermore, (r^2^−r_0_^2^)/r^2^ = −0.0060 (<0.1) and (r^2^−r_0′_^2^)/r^2^= 0.0115 (<0.1) The above parameters all met the external verification requirements [51,52], therefore, the constructed 3D-QSAR model has shown a satisfactory external prediction capability.

### 3.4. Determination of Substitution Characteristics Based on the Contour Maps

In the CoMFA model, the contributions of the steric and electrostatic fields are 68.10% and 31.90%, respectively, suggesting that the steric effect and electrical distribution of the groups will affect CI of QAs. In the steric field, the green area indicates that the introduction of bigger molecular groups in this area can improve the CI of QAs, while the yellow area indicates that the introduction of bigger molecular groups in this area can reduce the comprehensive effect of QAs. In the electrostatic field, the blue area indicates that the addition of positive groups is beneficial to improve the CI of QAs, and the red area indicates that the addition of negative groups is beneficial to the comprehensive effect of QAs.

**Figure 2:**
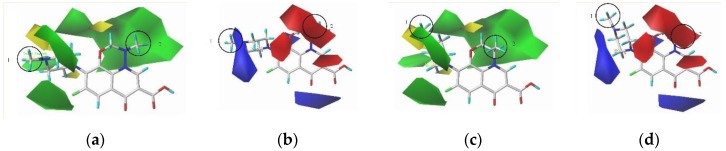
The contour maps of (**a**) Marbofloxacin (steric field), (**b**) marbofloxacin (electrostatic field), (**c**) levofloxacin (steric field), (**d**) levofloxacin (electrostatic field)

This study uses marbofloxacin and levofloxacin as examples to modify the substituents at positions 1 (CH_3_) and 2 (CH_3_), respectively. From the contour maps of marbofloxacin, it can be seen that the area near the 1-position substituent is mainly blue, indicating that the introduction of a positive group at the 1-position substituent is beneficial to improve the QAs’ CI; the green and red regions near the substituent at position 2 indicate that the introduction of bigger molecular groups and negative groups at the substituent in this position is conducive to improving the comprehensive effect value of the infrared and ultraviolet spectra of QAs. From the contour maps of levofloxacin, it can be seen that the vicinity of the substituent at position 1 is mainly blue, indicating that the introduction of a positively charged group at the substituent in this position is beneficial to improve the CI of QAs. The regions near the 2-position substituent are mainly green and red, indicating that the introduction of bigger molecular groups and negative groups at the 2-position substituent is conducive to improving the CI of QAs (Figure 2).

In summary, the 1- and 2-position substituents of marbofloxacin and levofloxacin were modified, respectively. Among them, positive groups (-H, -SiH_3_, -C_2_H_5_, -PH_2_, -C_3_H_7_, -C_4_H_9_, -C_5_H_11_) can be introduced at the 1-position substituent of marbofloxacin, and bigger molecular groups and negative groups (-CH_2_F, -C_2_H_3_, -C_2_H, -CH_2_OH, -NH_2_, -NO, -NO_2_, -CHO, -COOH) can be introduced at the 2-position substituent. A total of 13 single-substituted marbofloxacin derivatives and 57 double-substituted marbofloxacin derivatives. Positive groups (-H, -SiH_3_, -C_2_H_5_, -C_3_H_7_, -C_4_H_9_, -C_5_H_11_) can be introduced at the 1-position substituent of levofloxacin, and bigger molecular groups and negative charged group (-C_2_H_3_, -NO, -NO_2_, -OCH_3_, -OH, -CN) can be introduced at the 2-position substituent. A total of four single-substituted levofloxacin derivatives and 13 double-substituted levofloxacin derivatives. Thus, a total of 87 QA derivatives were generated. The molecular structures of these derivatives are shown in Table S1.

### 3.5. Evaluation of Molecular Spectral Characteristics, Functional Characteristics, Environmental Friendliness, and Stability of the QA Derivatives

#### 3.5.1. Molecular Spectral Characteristics

In this paper, the 3D-QSAR model was used to evaluate the CI of 87 QA derivatives. In addition, a single-effect model of infrared spectrum and ultraviolet spectrum was established in order to further verify the feasibility and rationality of the fuzzy matter-element method. Therefore, the single-effect model was used to evaluate the infrared spectrum intensity and ultraviolet spectrum single-effect of 87 QA derivatives (Table 6).

By analyzing the data in Table 6, it can be seen that when the comprehensive effects of 87 QA derivatives were enhanced, the single effects of the IRI and UVI were also enhanced. The average value of the comprehensive effect enhancement range was 186.46%, the average value of the single-effect enhancement amplitude of the infrared spectrum was 69.45%, and the average value of the single-effect enhancement amplitude of the ultraviolet spectrum was 1398.39%. Figure S1 was further generated to reflect the relationship between each QA structure and the associated activities (i.e., IRI, UVI, and CI) with marbofloxacin and levofloxacin used as the target molecules. Results indicated that the molecular design of QA derivatives showed positive effect on all activities with obvious enhancement of IRI, UVI, and CI values.

#### 3.5.2. The Enhancement of CI, IRI, and UVI of QA Derivatives’

##### The Quantitative Mechanism Analysis of the Spectral Enhancement of QA Derivatives Based on the Contour Map

**Figure 3:**
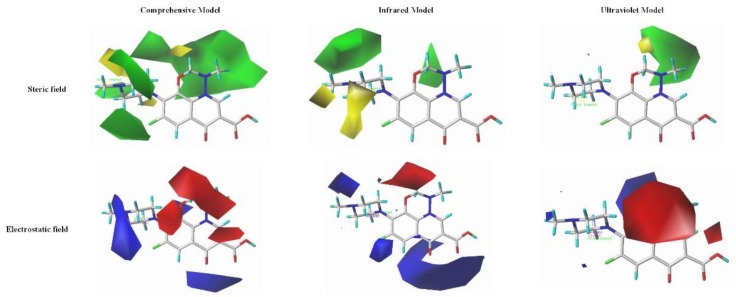
The contour maps of combined effect model and single effect model of infrared and ultraviolet spectra of marbofloxacin.

The contour map is used as the basis for obtaining accurate modification information in the 3D-QSAR model. Its spatial distribution and the size of each colored region are closely related to the activity of the designed derivative molecule. Therefore, this paper used marbofloxacin’s position 2 as an example to compare the combined effect model of the infrared and ultraviolet spectra with the contour map of the single-effect model. A qualitative mechanism analysis of the spectral enhancement of QA derivatives was conducted in order to reveal the effect of the modified information presented in the contour map on the degree of spectral enhancement of the derivative molecule (Figure 3).

According to the contour map of the steric field in the comprehensive effect model, the substituent at position 2 is surrounded by a green region, however, the other positions are also surrounded by green regions. Therefore, it can be considered that the steric field is not significant to the contribution of the 2-position substituent. In the infrared-effect model, there is no colored area near the 2-position substituent, thus, the steric field can be considered to have no contribution to the 2-position substituent in the model. In the ultraviolet-effect model, the green region is near the 2-position substituent, and it is the only green region distribution in the contour map. Therefore, the steric field is considered to have the most significant contribution to the 2-position substitution in the model. According to this, it can be qualitatively considered that the order of the contribution rate of the steric field to the modified position is UVI > CI > IRI.

According to the contour map of the steric field, the red area is distributed near the 2-position substituent; however, because the other positions are also close to red areas, the electrostatic field can be considered not significant to the contribution of the 2-position substituent. In the infrared-effect model, there is no colored area near the 2-position substituent, thus, the electrostatic field can be considered to have no contribution to the 2-position substituent in the model; in the ultraviolet-effect model, the red region is near the 2-position substituent, and it is the only red region distribution in the contour map. Therefore, the electrostatic field is considered to have the most significant contribution to the 2-position substitution in the ultraviolet-effect model. According to this, it can be qualitatively considered that the order of the contribution rate of the electrostatic field to the modified position is the UV single effect model > combined effect model > infrared single effect model.

In summary, according to the modified information provided by the contour map, it can be seen that the order of the contribution rate of the steric and electrostatic fields in the three groups of models are the UV single factor model > combined effect model > infrared single effect model. The results are consistent with spectral enhancement (the average value of the combined effect enhancement amplitude is 186.46%, the average value of the infrared spectrum single effect enhancement amplitude is 69.45%, and the ultraviolet spectrum single effect enhancement amplitude is 1398.39%). This shows that the spectral enhancement amplitude of QA derivatives has a certain internal relationship with the contribution of steric and electrostatic fields to its modified positions. The larger the contribution rate, the larger the corresponding spectral enhancement amplitude.

##### The Quantitative Mechanism Analysis of the Spectral Enhancement of QA Derivatives Based on the Modified Positions and the Properties of Substituted Groups

In addition to the distribution of colored regions in the contour map, the spectral enhancement amplitude is also affected by the properties of modified positions and the properties of substituted groups. Therefore, the number of modified positions, modified substitutes, and the properties of substituted groups were studied in this paper in order to reveal its intrinsic relationship with the spectral enhancement amplitude (Table 7).

According to the analysis of the data in Table 7, it was found that among the single substitution modification features, seven QA derivatives modified at the 1-position, such as Derivative 1, have an average enhancement range of the CI of 72.06%. The effect enhancement range is 60.02%, and the UV spectrum single effect enhancement range is 339.31%. Derivative 7 and the other 10 QA derivatives modified at the 2-position substituent have an average enhancement range of 113.08%. The single-effect enhancement of the infrared spectrum is 50.80%, and the single-effect enhancement of the ultraviolet spectrum is 421.09%. In the double-substitution modification feature, 70 QA derivatives, such as Derivative 14, are simultaneously modified at the 1- and 2-positions. The average enhancement of the CI is 208.39%, the IRI is 73.05%, and UVI is 1643.92%. From the results, it can be observed that under each substitution feature, the magnitude of the spectral enhancement amplitude in the three groups of models is still the UV single effect model > composite effect model > infrared single effect model, and the results are similar to the size distribution of the color regions in the contour map consistency.

In the comprehensive effect model, the spectral enhancement of QA derivatives obtained by modification at the 1-position, 2-position, and double substitution at 1- and 2-positions were 72.06%, 113.08%, and 208.39%, respectively. In the infrared single-effect model, the spectral enhancement of QA derivatives obtained by modification at the 1-position, 2-position, and double substitution at 1- and 2-positions were 60.02%, 50.80%, and 73.05%, respectively. In the effect model, the spectral enhancement of the QA derivatives obtained by modification at the 1-position, 2-position, and double substitution at 1- and 2-positions were 339.31%, 421.09%, and 1643.92%, respectively. By comparison, it can be found that, with the exception of the infrared single-effect model, the magnitudes of the spectral enhancement amplitudes in the remaining two models are both double-substitution features > 2-position substituent single-substitution features > 1-position substituent single-substitution features. In addition, the nature of the modified group selected for the 1-position substituent increased by 2.92%, the 2-position substituent was 61.93%, and the 1- and 2-position substituents were modified at the same time to 79.43%. The spectral enhancement amplitude has universal consistency, which indicates that the spectral enhancement amplitude of QA derivatives has a certain correlation with the properties of modification sites and groups. As the number of modification sites increases, the nature of the modified molecular groups becomes more prominent, and the spectrum will have a greater magnitude of enhancement.

#### 3.5.3. Molecular Stability Evaluation of QA Derivatives

The 87 QA derivatives designed based on the CoMFA model are used to characterize the stability of 87 QA derivatives in order to further derivatize QAs.

The positive frequency value of the molecule can directly reflect whether the molecule can stably exist in the environment. When the positive frequency value is greater than zero, it indicates that the molecule can exist stably in the environment; otherwise, it cannot [24,53]. In this paper, DFT was used to calculate the positive frequency values of 87 QAs derivatives to verify their stability (Table 8).

From the above results, the positive frequency values of the 87 QA derivatives are all greater than zero, indicating that all 87 QA derivatives designed in this study can stably exist in the environment.

#### 3.5.4. Functional and Environmental Friendliness Evaluation of QA derivatives

Zhao et al. [16,17] selected -lgLOEC (LOEC is the lowest observed effective concentration) to construct a hologram quantitative structure–activity relationship (HQSAR) model of the QAs genetic toxicity. QSAR models were designed independently to predict the genotoxicity, bioaccumulation, and biodegradability of the 87 QA derivatives (Table 9).

Genotoxicity refers to how QAs can selectively inhibit two enzymes that play a role in DNA synthesis in bacteria. Topoisomerase II and IV are two enzymes which interfere with the replication, transcription, repair, and recombination of bacterial DNA. These interferences make it impossible for bacteria to pass down genetic information, which lead to the increase in genotoxicity of QAs, making them conducive to improving the medicinal effect of this class of drugs [54]. According to the prediction results of genotoxicity, compared with marbofloxacin and levofloxacin, the genetic toxicity of 87 QA derivatives increased to varying degrees, and the increase range was 0.24%–29.01%.

The n-octanol-water partition coefficient (*K_ow_*) is one of the most important property parameters for studying the environmental behavior of organic matter. This parameter can simulate the distribution behavior of organic matter in lipid and water, and characterize the bioaccumulative ability of organic matter in environmental media. Organic substances with log*K_ow_* greater than 5.0 are banned worldwide [55]. According to the bioaccumulation prediction results, compared to marbofloxacin and levofloxacin, the bioaccumulation of 49 QA-derived molecules has been reduced to varying degrees, with a decrease of 0.25% to 60.47%, and the remaining 38 QA-derived molecules increased. The bioaccumulation of the molecule has increased to varying degrees, but its log*K_ow_* value is much lower than 5.0, which is below the banned threshold. Therefore, the bioaccumulation of all 87 QA derivatives are considered to be within the acceptable range.

The biodegradability is expressed by the LibDock Score (LDS) of the oxidoreductase enzyme of *Phanerochaete chrysosporium* and the QAs in the aerobic process of urban sewage treatment plants. The value of the LDS can represent the biodegradability of QAs [45]. Based on the prediction of biodegradability, it can be seen that compared to marbofloxacin and levofloxacin, the biodegradability of 34 QA derivatives has increased to varying degrees, with an increase of 0.12%–2.52%; for the remaining 53 QA derivatives, the biodegradability of biomolecules has decreased, with a decrease range of 0.12%–16.87%. Among them, the decline of nine QA derivatives is less than 1%, and their biodegradability can be considered unchanged. A total of 43 QA derivatives with increased or unchanged biodegradability can be selected.

In summary, among the 87 QA derivatives designed in this study, the properties of function (genetic toxicity) and environmentally friendliness (bioaccumulation, biodegradability) of 43 QAs are acceptable.

## 4. Conclusions

Based on DFT and fuzzy matter-element method, the CI of 24 quinolone antibiotics (QAs) were calculated in this study. A 3D-QSAR model was then constructed by using CI as the dependent variable. A total of 87 QA derivatives with enhanced CI and single effects were designed. All of the designed QA derivatives can stably exist in the environment, and the functionality and environmental friendliness of 43 QA derivatives are within the acceptable range. Derivative 3 may be considered as an alternative of marbofloxacin with increased genotoxicity (10.83%) and biodegradability (0.47%), and decreased bioaccumulation (24.83%). Derivative 74 with the improved genotoxicity (17.49%) and biodegradability (0.29%), as well as the decreased bioaccumulation (6.58%), shows a better performance than levofloxacin. In addition, molecules combined with the modification information provided by the contour map were analyzed to identify why the CI of QA derivatives was enhanced. The research output provides theoretical support for obtaining novel antibiotic drug molecules that are easy to detect and less harmful to the environment and human health.

## Figures and Tables

**Table 1:** Infrared spectral intensity (IRI) and ultraviolet spectral intensity (UVI) of 24 quinolone antibiotics (QAs).

No.	Name	IRI	UVI
Compound 1	Difloxacin	1924.76	2829.18
Compound 2	Enrofloxacin	1289.58	4394.34
Compound 3	Norfloxacin	1180.48	4627.19
Compound 4	Lomefloxacin	1788.98	1950.61
Compound 5	Ofloxacin	1428.91	3692.38
Compound 6	Pefloxacin	1587.11	3542.25
Compound 7	Fleroxacin	1882.42	1842.68
Compound 8	Ciprofloxacin	1247.65	4045.59
Compound 9	Balofloxacin	1368.19	3220.34
Compound 10	Marbofloxacin	1265.41	264.82
Compound 11	Pipemidic acid	2430.36	2563.72
Compound 12	Cinoxacin	1631.51	9680.14
Compound 13	Enoxacin	3427.59	8254.09
Compound 14	Gatifloxacin	1363.79	2835.67
Compound 15	Levofloxacin	1943.37	575.6
Compound 16	Rufloxacin	1015.14	437.85
Compound 17	Pazufloxacin	800.37	4259.61
Compound 18	Nadifloxacin	1623.96	2878.37
Compound 19	Sparfloxacin	1185.52	2542.11
Compound 20	Sarafloxacin	1539.67	2605.82
Compound 21	Besifloxacin	1682.72	3943.26
Compound 22	Clinafloxacin	1571.06	4875.36
Compound 23	Grepafloxacin	1489.4	2390.87
Compound 24	Temafloxacin	2155.95	8643.35

**Table 2:** The results of fuzzy membership, standard fuzzy matter-element and variance compound fuzzy matter-element matrix QAs.

*M_i_*	*R’_mn_*		*R_Δ_*		Normalization	
	*C*_1_	*C*_2_	*C*_1_	*C*_2_	*C*_1_	*C*_2_
*M*_1_	0.5615	0.2923	0.1922	0.5009	0.4280	0.2724
*M*_2_	0.3762	0.4540	0.3891	0.2982	0.1862	0.4386
*M*_3_	0.3444	0.4780	0.4298	0.2725	0.1447	0.4633
*M*_4_	0.5219	0.2015	0.2285	0.6376	0.3763	0.1790
*M*_5_	0.4169	0.3814	0.3400	0.3826	0.2392	0.3640
*M*_6_	0.4630	0.3659	0.2883	0.4020	0.2995	0.3481
*M*_7_	0.5492	0.1904	0.2032	0.6555	0.4119	0.1676
*M*_8_	0.3640	0.4179	0.4045	0.3388	0.1702	0.4016
*M*_9_	0.3992	0.3327	0.3610	0.4453	0.2161	0.3139
*M*_10_	0.3692	0.0274	0.3979	0.9460	0.1770	0.0000
*M*_11_	0.7091	0.2648	0.0846	0.5405	0.6204	0.2442
*M*_12_	0.4760	1.0000	0.2746	0.0000	0.3164	1.0000
*M*_13_	1.0000	0.8527	0.0000	0.0217	1.0000	0.8485
*M*_14_	0.3979	0.2929	0.3625	0.4999	0.2145	0.2730
*M*_15_	0.5670	0.0595	0.1875	0.8846	0.4351	0.0330
*M*_16_	0.2962	0.0452	0.4954	0.9116	0.0817	0.0184
*M*_17_	0.2335	0.4400	0.5875	0.3136	0.0000	0.4243
*M*_18_	0.4738	0.2973	0.2769	0.4937	0.3135	0.2776
*M*_19_	0.3459	0.2626	0.4279	0.5437	0.1466	0.2419
*M*_20_	0.4492	0.2692	0.3034	0.5341	0.2814	0.2486
*M*_21_	0.4909	0.4074	0.2591	0.3512	0.3358	0.3907
*M*_22_	0.4584	0.5036	0.2934	0.2464	0.2933	0.4897
*M*_23_	0.4345	0.2470	0.3198	0.5670	0.2623	0.2258
*M*_24_	0.6290	0.8929	0.1376	0.0115	0.5160	0.8899

**Table 3:** The results of objective weightings and comprehensive weightings of QAs’ infrared and ultraviolet spectra.

Entropy		Entropy Weights		Comprehensive Weights	
*S*_1_	*S*_2_	*W*_1_	*W*_2_	*W’*_1_	*W’*_2_
−0.9966	−0.9950	0.5002	0.4998	0.5001	0.4999

From the results, it can be seen that the objective weights calculated by the entropy method are 0.5002 and 0.4998, and the comprehensive weights are 0.5001 and 0.4999, which are close to 0.5.

**Table 4:** Calculation of comprehensive effects of infrared and ultraviolet spectra of 24 QAs based on fuzzy matter-element method.

*e*_i_	Comprehensive Effects	*e*_i_	Comprehensive Effects	*e*_i_	Comprehensive Effects
*e*_1_	0.4113	*e*_9_	0.3651	*e*_17_	0.3288
*e*_2_	0.4138	*e*_10_	0.1803	*e*_18_	0.3793
*e*_3_	0.4074	*e*_11_	0.4409	*e*_19_	0.3030
*e*_4_	0.3419	*e*_12_	0.6295	*e*_20_	0.3529
*e*_5_	0.3989	*e*_13_	0.8958	*e*_21_	0.4476
*e*_6_	0.4125	*e*_14_	0.3433	*e*_22_	0.4805
*e*_7_	0.3447	*e*_15_	0.2678	*e*_23_	0.3341
*e*_8_	0.3904	*e*_16_	0.1613	*e*_24_	0.7269

**Table 5:** The predicted combined effects of infrared and ultraviolet spectra (CI) of QAs using Comparative Molecular Field Analysis (CoMFA) model.

No.	Calculated Value	Predicted Value	Relative Deviation
^a^ Compound 3	0.4074	0.3990	2.06%
^a^ Compound 4	0.3419	0.3540	−3.54%
^b^ Compound 7	0.3447	0.3970	−15.17%
^a^ Compound 8	0.3904	0.4170	−6.81%
^a^ Compound 9	0.3651	0.3620	0.85%
^a^ Compound 10	0.1803	0.1630	9.60%
^a^ Compound 11	0.4409	0.4430	−0.48%
^a^ Compound 12	0.6295	0.6520	−3.57%
^a^ Compound 15	0.2678	0.2680	−0.07%
^a^ Compound 16	0.1613	0.1950	−20.89%
^b^ Compound 18	0.3793	0.3800	−0.18%
^b^ Compound 20	0.3529	0.3770	−6.83%
^a^ Compound 21	0.4476	0.4300	3.93%
^a^ Compound 22	0.4805	0.4390	8.64%
^a^ Compound 23	0.3341	0.3290	1.53%
^a,b^ Compound 24	0.7269	0.7240	0.40%

^a^ Training set; ^b^ Test set.

**Table 6:** The predicted evaluation of 87 QA derivatives’ molecular spectral characteristic.

QA Derivatives	Position of Substitution		CI	Relative Deviation	IRI	Relative Deviation	UVI	Relative Deviation
	1-Position	2-Position						
Marbofloxacin	CH_3_	CH_3_	0.1803		1265.4100		264.8200	
Derivative 1	SiH_3_	-	0.2820	56.41%	2046.4446	61.72%	920.4496	247.58%
Derivative 2	C_2_H_5_	-	0.2690	49.20%	2128.1390	68.18%	749.8942	183.17%
Derivative 3	PH_2_	-	0.2850	58.07%	2051.1622	62.09%	916.2205	245.98%
Derivative 4	C_3_H_7_	-	0.2690	49.20%	2113.4890	67.02%	711.2135	168.56%
Derivative 5	C_4_H_9_	-	0.2690	49.20%	2133.0449	68.57%	714.4963	169.80%
Derivative 6	C_5_H_11_	-	0.2690	49.20%	2133.0449	68.57%	712.8530	169.18%
Derivative 7	-	CH_2_F	0.3510	94.68%	2051.1622	62.09%	1492.7944	463.70%
Derivative 8	-	C_2_H_3_	0.3210	78.04%	2023.0192	59.87%	1205.0359	355.04%
Derivative 9	-	C_2_H	0.3100	71.94%	1976.9696	56.23%	805.3784	204.12%
Derivative 10	-	CH_2_OH	0.2930	62.51%	2013.7242	59.14%	972.7472	267.32%
Derivative 11	-	NH_2_	0.3030	68.05%	2051.1622	62.09%	629.5062	137.71%
Derivative 12	-	NO	0.3530	95.78%	2009.0928	58.77%	1061.6956	300.91%
Derivative 13	-	NO_2_	0.3590	99.11%	1995.2623	57.68%	1142.8783	331.57%
Derivative 14	SiH_3_	CH_2_F	0.3550	96.89%	2060.6299	62.84%	1442.1154	444.56%
Derivative 15	PH_2_	CH_2_F	0.3510	94.68%	2046.4446	61.72%	1492.7944	463.70%
Derivative 16	C_3_H_7_	CH_2_F	0.8050	346.48%	2511.8864	98.50%	8729.7137	3196.47%
Derivative 17	C_4_H_9_	CH_2_F	0.8060	347.03%	2511.8864	98.50%	8729.7137	3196.47%
Derivative 18	C_5_H_11_	CH_2_F	0.8060	347.03%	2511.8864	98.50%	8729.7137	3196.47%
Derivative 19	H	C_2_H_3_	0.8150	352.02%	2624.2185	107.38%	9571.9407	3514.51%
Derivative 20	SiH_3_	C_2_H_3_	0.8210	355.35%	2630.2680	107.86%	9354.0567	3432.23%
Derivative 21	C_2_H_5_	C_2_H_3_	0.8190	354.24%	2630.2680	107.86%	9332.5430	3424.11%
Derivative 22	PH_2_	C_2_H_3_	0.8180	353.69%	2630.2680	107.86%	9506.0479	3489.63%
Derivative 23	C_3_H_7_	C_2_H_3_	0.8210	355.35%	2636.3314	108.34%	8356.0302	3055.36%
Derivative 24	C_4_H_9_	C_2_H_3_	0.8210	355.35%	2636.3314	108.34%	8298.5077	3033.64%
Derivative 25	C_5_H_11_	C_2_H_3_	0.8210	355.35%	2654.6056	109.78%	7870.4579	2872.00%
Derivative 26	H	C_2_H	0.8160	352.58%	2570.3958	103.13%	9549.9259	3506.20%
Derivative 27	SiH_3_	C_2_H	0.8210	355.35%	2594.1794	105.01%	9141.1324	3351.83%
Derivative 28	C_2_H_5_	C_2_H	0.8210	355.35%	2612.1614	106.43%	8974.2879	3288.83%
Derivative 29	PH_2_	C_2_H	0.8170	353.13%	2588.2129	104.54%	9484.1846	3481.37%
Derivative 30	C_3_H_7_	C_2_H	0.7960	341.49%	2588.2129	104.54%	6180.1640	2233.72%
Derivative 31	C_4_H_9_	C_2_H	0.7960	341.49%	2588.2129	104.54%	5807.6442	2093.05%
Derivative 32	C_5_H_11_	C_2_H	0.7950	340.93%	2588.2129	104.54%	5754.3994	2072.95%
Derivative 33	H	CH_2_OH	0.8200	354.80%	2523.4808	99.42%	9660.5088	3547.95%
Derivative 34	SiH_3_	CH_2_OH	0.8260	358.13%	2535.1286	100.34%	9397.2331	3448.54%
Derivative 35	C_2_H_5_	CH_2_OH	0.8250	357.57%	2535.1286	100.34%	8749.8378	3204.07%
Derivative 36	PH_2_	CH_2_OH	0.8220	355.91%	2529.2980	99.88%	9616.1228	3531.19%
Derivative 37	C_3_H_7_	CH_2_OH	0.8230	356.46%	2523.4808	99.42%	8933.0548	3273.26%
Derivative 38	C_4_H_9_	CH_2_OH	0.8230	356.46%	2529.2980	99.88%	8974.2879	3288.83%
Derivative 39	C_5_H_11_	CH_2_OH	0.8230	356.46%	2529.2980	99.88%	8974.2879	3288.83%
Derivative 40	H	NH_2_	0.7920	339.27%	2546.8303	101.27%	8609.9375	3151.24%
Derivative 41	SiH_3_	NH_2_	0.8000	343.70%	2558.5859	102.19%	8317.6377	3040.86%
Derivative 42	C_2_H_5_	NH_2_	0.8020	344.81%	2582.2602	104.07%	8336.8118	3048.11%
Derivative 43	PH_2_	NH_2_	0.8010	344.26%	2582.2602	104.07%	8336.8118	3048.11%
Derivative 44	C_3_H_7_	NH_2_	0.7720	328.18%	2594.1794	105.01%	6123.5039	2212.33%
Derivative 45	C_4_H_9_	NH_2_	0.3450	91.35%	2162.7185	70.91%	2805.4336	959.37%
Derivative 46	C_5_H_11_	NH_2_	0.3730	106.88%	2187.7616	72.89%	2792.5438	954.51%
Derivative 47	H	NO	0.3200	77.48%	2137.9621	68.95%	1606.9413	506.81%
Derivative 48	SiH_3_	NO	0.2730	51.41%	2192.8049	73.29%	1166.8096	340.60%
Derivative 49	C_2_H_5_	NO	0.2420	34.22%	2157.7444	70.52%	984.0111	271.58%
Derivative 50	PH_2_	NO	0.4150	130.17%	1972.4227	55.87%	2074.9135	683.52%
Derivative 51	C_3_H_7_	NO	0.2440	35.33%	2128.1390	68.18%	990.8319	274.15%
Derivative 52	C_4_H_9_	NO	0.2460	36.44%	2137.9621	68.95%	933.2543	252.41%
Derivative 53	C_5_H_11_	NO	0.2460	36.44%	2128.1390	68.18%	937.5620	254.04%
Derivative 54	H	NO_2_	0.3170	75.82%	2079.6967	64.35%	1534.6170	479.49%
Derivative 55	SiH_3_	NO_2_	0.2530	40.32%	2113.4890	67.02%	1156.1122	336.57%
Derivative 56	C_2_H_5_	NO_2_	0.2340	29.78%	2094.1125	65.49%	1324.3415	400.09%
Derivative 57	C_3_H_7_	NO_2_	0.2350	30.34%	2074.9135	63.97%	918.3326	246.78%
Derivative 58	C_4_H_9_	NO_2_	0.2360	30.89%	2089.2961	65.11%	889.2011	235.78%
Derivative 59	C_5_H_11_	NO_2_	0.2360	30.89%	2089.2961	65.11%	870.9636	228.89%
Derivative 60	H	CHO	0.4500	149.58%	1981.5270	56.59%	3019.9517	1040.38%
Derivative 61	SiH_3_	CHO	0.4110	127.95%	1896.7059	49.89%	2488.8573	839.83%
Derivative 62	C_2_H_5_	CHO	0.3270	81.36%	1909.8533	50.93%	1753.8805	562.29%
Derivative 63	C_3_H_7_	CHO	0.3130	73.60%	1918.6687	51.62%	1774.1895	569.96%
Derivative 64	C_4_H_9_	CHO	0.3260	80.81%	1927.5249	52.32%	2032.3570	667.45%
Derivative 65	C_5_H_11_	CHO	0.3270	81.36%	1923.0917	51.97%	2018.3664	662.17%
Derivative 66	H	COOH	0.4550	152.36%	2065.3802	63.22%	2951.2092	1014.42%
Derivative 67	SiH_3_	COOH	0.3960	119.63%	2074.9135	63.97%	2301.4418	769.06%
Derivative 68	C_2_H_5_	COOH	0.3170	75.82%	1995.2623	57.68%	1923.0917	626.19%
Derivative 69	C_3_H_7_	COOH	0.3030	68.05%	1986.0949	56.95%	1905.4607	619.53%
Derivative 70	C_4_H_9_	COOH	0.3130	73.60%	1986.0949	56.95%	2108.6281	696.25%
Levofloxacin	CH_3_	CH_3_	0.2678		1943.3700		575.6000	
Derivative 71	C_4_H_9_		0.7850	193.13%	2409.9054	24.01%	7430.1914	1190.86%
Derivative 72	C_4_H_9_	C_2_H_3_	0.7500	180.06%	2636.3314	35.66%	5834.4510	913.63%
Derivative 73	C_3_H_7_	OH	0.7430	177.45%	2552.7013	31.35%	2108.6281	266.34%
Derivative 74	C_3_H_7_	OH	0.6690	149.81%	2328.0913	19.80%	1741.8069	202.61%
Derivative 75	H	OH	0.7210	169.23%	2404.3628	23.72%	3881.5037	574.34%
Derivative 76		OCH_3_	0.7670	186.41%	2488.8573	28.07%	7328.2453	1173.15%
Derivative 77	C_2_H_5_	OCH_3_	0.7420	177.07%	2624.2185	35.03%	5296.6344	820.19%
Derivative 78	SiH_3_	OCH_3_	0.7560	182.30%	2529.2980	30.15%	7533.5556	1208.82%
Derivative 79	C_5_H_11_	OCH_3_	0.7560	182.30%	2477.4221	27.48%	1496.2357	159.94%
Derivative 80	C_3_H_7_	CN	0.7550	181.93%	2618.1830	34.72%	6095.3690	958.96%
Derivative 81	SiH_3_	NO	0.7560	182.30%	2275.0974	17.07%	3169.5675	450.65%
Derivative 82		NO_2_	0.7530	181.18%	2642.4088	35.97%	5035.0061	774.74%
Derivative 83	C_2_H_5_	NO_2_	0.7590	183.42%	2466.0393	26.89%	5223.9619	807.57%
Derivative 84	SiH_3_	NO_2_	0.7680	186.78%	2387.8113	22.87%	7177.9429	1147.04%
Derivative 85	C_3_H_7_	NO_2_	0.7530	181.18%	2506.1093	28.96%	5105.0500	786.91%
Derivative 86	C_4_H_9_	NO_2_	0.7540	181.55%	2588.2129	33.18%	5495.4087	854.73%
Derivative 87		CHO	0.7850	193.13%	2488.8573	28.07%	1741.8069	202.61%

**Table 7:** The modified positions and properties of 87 QAs Derivatives.

Molecular Substitution Type	QA Derivatives				Modified Positions and Properties of Substituted Groups				
		1-Position Positive	Relative Deviation	2-Position Negative	Relative Deviation	2-Position Bigger Group	Relative Deviation	2-Position Coupling	1, 2-Position Coupling
	Marbofloxacin/Levofloxacin	2.331	-	2.331	-	15	-	-	-
Monosubstitution	Derivative 1	2.120	9.05%	-	-	-	-	-	-
	Derivative 2	2.315	0.69%	-	-	-	-	-	-
	Derivative 3	2.154	7.59%	-	-	-	-	-	-
	Derivative 4	2.314	0.73%	-	-	-	-	-	-
	Derivative 5	2.313	0.77%	-	-	-	-	-	-
	Derivative 6	2.312	0.82%	-	-	-	-	-	-
	Derivative 71	2.313	0.77%	-	-	-	-	-	-
	Derivative 7	-	-	2.644	13.43%	33	120.00%	66.71%	-
	Derivative 8	-	-	2.358	1.16%	27	80.00%	40.58%	-
	Derivative 9	-	-	2.530	8.54%	25	66.67%	37.60%	-
	Derivative 10	-	-	2.491	6.86%	31	106.67%	56.77%	-
	Derivative 11	-	-	2.437	4.55%	16	6.67%	5.61%	-
	Derivative 12	-	-	2.920	25.27%	30	100.00%	62.63%	-
	Derivative 13	-	-	3.104	33.16%	46	206.67%	119.91%	-
	Derivative 76	-	-	2.460	5.53%	31	106.67%	56.10%	-
	Derivative 82	-	-	3.104	33.16%	46	206.67%	119.91%	-
	Derivative 87	-	-	2.647	13.56%	29	93.33%	53.44%	-
Disubstituted	Derivative 14	2.120	9.05%	2.644	13.43%	33	120.00%	85.90%	94.95%
	Derivative 15	2.154	7.59%	2.644	13.43%	33	120.00%	85.90%	93.49%
	Derivative 16	2.314	0.73%	2.644	13.43%	33	120.00%	85.90%	86.63%
	Derivative 17	2.313	0.77%	2.644	13.43%	33	120.00%	85.90%	86.67%
	Derivative 18	2.312	0.82%	2.644	13.43%	33	120.00%	85.90%	86.71%
	Derivative 19	2.200	5.62%	2.358	1.16%	27	80.00%	54.77%	60.39%
	Derivative 20	2.120	9.05%	2.358	1.16%	27	80.00%	54.77%	63.82%
	Derivative 21	2.315	0.69%	2.358	1.16%	27	80.00%	54.77%	55.46%
	Derivative 22	2.154	7.59%	2.358	1.16%	27	80.00%	54.77%	62.36%
	Derivative 23	2.314	0.73%	2.358	1.16%	27	80.00%	54.77%	55.50%
	Derivative 24	2.313	0.77%	2.358	1.16%	27	80.00%	54.77%	55.54%
	Derivative 25	2.312	0.82%	2.358	1.16%	27	80.00%	54.77%	55.59%
	Derivative 26	2.200	5.62%	2.530	8.54%	25	66.67%	48.07%	53.69%
	Derivative 27	2.120	9.05%	2.530	8.54%	25	66.67%	48.07%	57.12%
	Derivative 28	2.315	0.69%	2.530	8.54%	25	66.67%	48.07%	48.75%
	Derivative 29	2.154	7.59%	2.530	8.54%	25	66.67%	48.07%	55.66%
	Derivative 30	2.314	0.73%	2.530	8.54%	25	66.67%	48.07%	48.79%
	Derivative 31	2.313	0.77%	2.530	8.54%	25	66.67%	48.07%	48.84%
	Derivative 32	2.312	0.82%	2.530	8.54%	25	66.67%	48.07%	48.88%
	Derivative 33	2.200	5.62%	2.491	6.86%	31	106.67%	74.73%	80.35%
	Derivative 34	2.120	9.05%	2.491	6.86%	31	106.67%	74.73%	83.78%
	Derivative 35	2.315	0.69%	2.491	6.86%	31	106.67%	74.73%	75.42%
	Derivative 36	2.154	7.59%	2.491	6.86%	31	106.67%	74.73%	82.32%
	Derivative 37	2.314	0.73%	2.491	6.86%	31	106.67%	74.73%	75.46%
	Derivative 38	2.313	0.77%	2.491	6.86%	31	106.67%	74.73%	75.50%
	Derivative 39	2.312	0.82%	2.491	6.86%	31	106.67%	74.73%	75.54%
	Derivative 40	2.200	5.62%	2.437	4.55%	16	6.67%	5.99%	11.61%
	Derivative 41	2.120	9.05%	2.437	4.55%	16	6.67%	5.99%	15.04%
	Derivative 42	2.315	0.69%	2.437	4.55%	16	6.67%	5.99%	6.67%
	Derivative 43	2.154	7.59%	2.437	4.55%	16	6.67%	5.99%	13.58%
	Derivative 44	2.314	0.73%	2.437	4.55%	16	6.67%	5.99%	6.72%
	Derivative 45	2.313	0.77%	2.437	4.55%	16	6.67%	5.99%	6.76%
	Derivative 46	2.312	0.82%	2.437	4.55%	16	6.67%	5.99%	6.80%
	Derivative 47	2.200	5.62%	2.920	25.27%	30	100.00%	76.09%	81.71%
	Derivative 48	2.120	9.05%	2.920	25.27%	30	100.00%	76.09%	85.14%
	Derivative 49	2.315	0.69%	2.920	25.27%	30	100.00%	76.09%	76.77%
	Derivative 50	2.154	7.59%	2.920	25.27%	30	100.00%	76.09%	83.68%
	Derivative 51	2.314	0.73%	2.920	25.27%	30	100.00%	76.09%	76.82%
	Derivative 52	2.313	0.77%	2.920	25.27%	30	100.00%	76.09%	76.86%
	Derivative 53	2.312	0.82%	2.920	25.27%	30	100.00%	76.09%	76.90%
	Derivative 54	2.200	5.62%	3.104	33.16%	46	206.67%	151.15%	156.76%
	Derivative 55	2.120	9.05%	3.104	33.16%	46	206.67%	151.15%	160.20%
	Derivative 56	2.315	0.69%	3.104	33.16%	46	206.67%	151.15%	151.83%
	Derivative 57	2.314	0.73%	3.104	33.16%	46	206.67%	151.15%	151.87%
	Derivative 58	2.313	0.77%	3.104	33.16%	46	206.67%	151.15%	151.92%
	Derivative 59	2.312	0.82%	3.104	33.16%	46	206.67%	151.15%	151.96%
	Derivative 60	2.200	5.62%	2.647	13.56%	29	93.33%	67.80%	73.42%
	Derivative 61	2.120	9.05%	2.647	13.56%	29	93.33%	67.80%	76.86%
	Derivative 62	2.315	0.69%	2.647	13.56%	29	93.33%	67.80%	68.49%
	Derivative 63	2.314	0.73%	2.647	13.56%	29	93.33%	67.80%	68.53%
	Derivative 64	2.313	0.77%	2.647	13.56%	29	93.33%	67.80%	68.58%
	Derivative 65	2.312	0.82%	2.647	13.56%	29	93.33%	67.80%	68.62%
	Derivative 66	2.200	5.62%	2.769	18.79%	45	200.00%	142.01%	147.63%
	Derivative 67	2.120	9.05%	2.769	18.79%	45	200.00%	142.01%	151.06%
	Derivative 68	2.315	0.69%	2.769	18.79%	45	200.00%	142.01%	142.70%
	Derivative 69	2.314	0.73%	2.769	18.79%	45	200.00%	142.01%	142.74%
	Derivative 70	2.313	0.77%	2.769	18.79%	45	200.00%	142.01%	142.79%
	Derivative 72	2.313	0.77%	2.358	1.16%	27	80.00%	54.77%	55.54%
	Derivative 73	2.314	0.73%	2.585	10.90%	17	13.33%	12.55%	13.28%
	Derivative 74	2.314	0.73%	2.585	10.90%	17	13.33%	12.55%	13.28%
	Derivative 75	2.200	5.62%	2.585	10.90%	17	13.33%	12.55%	18.17%
	Derivative 77	2.315	0.69%	2.460	5.53%	31	106.67%	74.30%	74.99%
	Derivative 78	2.120	9.05%	2.460	5.53%	31	106.67%	74.30%	83.36%
	Derivative 79	2.312	0.82%	2.460	5.53%	31	106.67%	74.30%	75.12%
	Derivative 80	2.314	0.73%	2.792	19.78%	26	73.33%	56.20%	56.92%
	Derivative 81	2.120	9.05%	2.920	25.27%	30	100.00%	76.09%	85.14%
	Derivative 83	2.315	0.69%	3.104	33.16%	46	206.67%	151.15%	151.83%
	Derivative 84	2.120	9.05%	3.104	33.16%	46	206.67%	151.15%	160.20%
	Derivative 85	2.314	0.73%	3.104	33.16%	46	206.67%	151.15%	151.87%
	Derivative 86	2.313	0.77%	3.104	33.16%	46	206.67%	151.15%	151.92%

**Table 8:** The results of positive frequency values of 87 QAs derivatives.

QA Derivatives	Positive Frequency Value	QA Derivatives	Positive Frequency Value	QA Derivatives	Positive Frequency Value
Derivative 1	20.78	Derivative 30	14.97	Derivative 59	10.15
Derivative 2	17.07	Derivative 31	14.85	Derivative 60	28.78
Derivative 3	19.93	Derivative 32	12.63	Derivative 61	18.22
Derivative 4	15.47	Derivative 33	24.17	Derivative 62	25.68
Derivative 5	14.17	Derivative 34	17.19	Derivative 63	23.33
Derivative 6	13.15	Derivative 35	18.39	Derivative 64	21.80
Derivative 7	25.16	Derivative 36	19.52	Derivative 65	18.79
Derivative 8	21.84	Derivative 37	19.74	Derivative 66	28.44
Derivative 9	22.60	Derivative 38	14.32	Derivative 67	23.12
Derivative 10	20.75	Derivative 39	13.44	Derivative 68	24.75
Derivative 11	21.58	Derivative 40	31.02	Derivative 69	22.49
Derivative 12	24.54	Derivative 41	20.39	Derivative 70	20.99
Derivative 13	22.29	Derivative 42	17.77	Derivative 71	15.37
Derivative 14	20.67	Derivative 43	20.83	Derivative 72	11.09
Derivative 15	20.88	Derivative 44	20.82	Derivative 73	15.30
Derivative 16	18.33	Derivative 45	15.87	Derivative 74	16.74
Derivative 17	17.08	Derivative 46	12.92	Derivative 75	28.59
Derivative 18	14.97	Derivative 47	24.63	Derivative 76	21.50
Derivative 19	21.27	Derivative 48	19.56	Derivative 77	16.28
Derivative 20	16.15	Derivative 49	21.39	Derivative 78	19.62
Derivative 21	19.96	Derivative 50	14.89	Derivative 79	9.36
Derivative 22	18.43	Derivative 51	13.37	Derivative 80	18.66
Derivative 23	16.27	Derivative 52	12.78	Derivative 81	16.26
Derivative 24	15.53	Derivative 53	7.10	Derivative 82	21.73
Derivative 25	14.51	Derivative 54	23.28	Derivative 83	20.00
Derivative 26	24.80	Derivative 55	20.02	Derivative 84	21.43
Derivative 27	18.49	Derivative 56	17.86	Derivative 85	18.49
Derivative 28	21.60	Derivative 57	13.65	Derivative 86	11.53
Derivative 29	20.14	Derivative 58	12.72	Derivative 87	23.13

**Table 9:** Predicted results of genotoxicity, bioaccumulation, and biodegradability of 87 QA derivatives.

QA Derivatives	Genotoxicity	Relative Deviation	Bioaccumulation	Relative Deviation	Biodegradability	Relative Deviation
Marbofloxacin	8.4600		1.1840		1.7050	
Derivative 1	9.5390	12.75%	0.9650	−18.50%	1.7070	0.12%
Derivative 2	9.8690	16.65%	1.1630	−1.77%	1.7250	1.17%
Derivative 3	9.3760	10.83%	0.8900	−24.83%	1.7130	0.47%
Derivative 4	9.6020	13.50%	1.1730	−0.93%	1.7240	1.11%
Derivative 5	9.5320	12.67%	1.1740	−0.84%	1.7250	1.17%
Derivative 6	9.6380	13.92%	1.1750	−0.76%	1.7240	1.11%
Derivative 7	9.0310	6.75%	1.3610	14.95%	1.7190	0.82%
Derivative 8	8.9180	5.41%	1.3730	15.96%	1.7290	1.41%
Derivative 9	8.5310	0.84%	1.2810	8.19%	1.7170	0.70%
Derivative 10	9.2950	9.87%	1.5920	34.46%	1.7030	−0.12%
Derivative 11	9.8810	16.80%	1.1110	−6.17%	1.7130	0.47%
Derivative 12	8.4810	0.25%	1.5260	28.89%	1.6900	−0.88%
Derivative 13	9.0470	6.94%	1.2460	5.24%	1.7350	1.76%
Derivative 14	9.6010	13.49%	1.1590	−2.11%	1.7160	0.65%
Derivative 15	9.4670	11.90%	1.0520	−11.15%	1.7190	0.82%
Derivative 16	9.8060	15.91%	0.6090	−48.56%	1.4940	−12.38%
Derivative 17	9.7360	15.08%	0.6080	−48.65%	1.4940	−12.38%
Derivative 18	9.8420	16.34%	0.6060	−48.82%	1.4940	−12.38%
Derivative 19	9.2230	9.02%	0.5660	−52.20%	1.5050	−11.73%
Derivative 20	9.4870	12.14%	0.5650	−52.28%	1.4990	−12.08%
Derivative 21	9.9670	17.81%	0.5720	−51.69%	1.5000	−12.02%
Derivative 22	9.4730	11.97%	0.5430	−54.14%	1.5040	−11.79%
Derivative 23	9.7000	14.66%	0.5760	−51.35%	1.5070	−11.61%
Derivative 24	9.6300	13.83%	0.5760	−51.35%	1.5060	−11.67%
Derivative 25	9.7350	15.07%	0.5760	−51.35%	1.5100	−11.44%
Derivative 26	8.8350	4.43%	0.5470	−53.80%	1.5260	−10.50%
Derivative 27	8.9990	6.37%	0.5450	−53.97%	1.5230	−10.67%
Derivative 28	9.5790	13.23%	0.5550	−53.13%	1.5290	−10.32%
Derivative 29	9.0860	7.40%	0.5230	−55.83%	1.5220	−10.73%
Derivative 30	9.3130	10.08%	0.5910	−50.08%	1.5270	−10.44%
Derivative 31	9.2430	9.26%	0.5950	−49.75%	1.5290	−10.32%
Derivative 32	9.3480	10.50%	0.5970	−49.58%	1.5300	−10.26%
Derivative 33	9.6000	13.48%	0.5350	−54.81%	1.5060	−11.67%
Derivative 34	9.7820	15.63%	0.5330	−54.98%	1.5010	−11.96%
Derivative 35	10.3440	22.27%	0.5450	−53.97%	1.5030	−11.85%
Derivative 36	9.8510	16.44%	0.5120	−56.76%	1.5020	−11.91%
Derivative 37	10.0780	19.13%	0.5440	−54.05%	1.4980	−12.14%
Derivative 38	10.0070	18.29%	0.5440	−54.05%	1.4990	−12.08%
Derivative 39	10.1130	19.54%	0.5440	−54.05%	1.5010	−11.96%
Derivative 40	10.1700	20.21%	0.5020	−57.60%	1.5130	−11.26%
Derivative 41	10.1300	19.74%	0.4950	−58.19%	1.5120	−11.32%
Derivative 42	10.9140	29.01%	0.5150	−56.50%	1.5090	−11.50%
Derivative 43	10.4210	23.18%	0.4680	−60.47%	1.5150	−11.14%
Derivative 44	10.6480	25.86%	0.5600	−52.70%	1.5180	−10.97%
Derivative 45	10.5810	25.07%	1.1810	−0.25%	1.7350	1.76%
Derivative 46	10.6870	26.32%	1.1840	0.00%	1.7340	1.70%
Derivative 47	8.7900	3.90%	1.4680	23.99%	1.7140	0.53%
Derivative 48	8.8820	4.99%	1.5270	28.97%	1.7120	0.41%
Derivative 49	9.5340	12.70%	1.6600	40.20%	1.7140	0.53%
Derivative 50	10.2130	20.72%	1.2030	1.60%	1.7480	2.52%
Derivative 51	9.2680	9.55%	1.6710	41.13%	1.7140	0.53%
Derivative 52	9.1970	8.71%	1.6780	41.72%	1.7150	0.59%
Derivative 53	9.3030	9.96%	1.6730	41.30%	1.7150	0.59%
Derivative 54	9.3480	10.50%	1.2920	9.12%	1.7150	0.59%
Derivative 55	9.5060	12.36%	1.2600	6.42%	1.7170	0.70%
Derivative 56	10.0920	19.29%	1.5080	27.36%	1.7190	0.82%
Derivative 57	9.8260	16.15%	1.5030	26.94%	1.7150	0.59%
Derivative 58	9.7550	15.31%	1.5080	27.36%	1.7150	0.59%
Derivative 59	9.8610	16.56%	1.5100	27.53%	1.7160	0.65%
Derivative 60	8.7220	3.10%	0.8730	−26.27%	1.6940	−0.65%
Derivative 61	9.2850	9.75%	0.9190	−22.38%	1.6930	−0.70%
Derivative 62	9.4710	11.95%	1.1430	−3.46%	1.7000	−0.29%
Derivative 63	9.1970	8.71%	1.1680	−1.35%	1.7000	−0.29%
Derivative 64	9.1270	7.88%	1.1810	−0.25%	1.7020	−0.18%
Derivative 65	9.2330	9.14%	1.1870	0.25%	1.7000	−0.29%
Derivative 66	8.8390	4.48%	1.2240	3.38%	1.7380	1.94%
Derivative 67	9.4460	11.65%	1.2440	5.07%	1.7430	2.23%
Derivative 68	9.5880	13.33%	1.5060	27.20%	1.7430	2.23%
Derivative 69	9.3150	10.11%	1.5320	29.39%	1.7400	2.05%
Derivative 70	9.2440	9.27%	1.5460	30.57%	1.7350	1.76%
Levofloxacin	7.9750		1.4590		1.7070	
Derivative 71	8.9340	12.03%	1.5280	4.73%	1.4730	−13.71%
Derivative 72	8.7350	9.53%	1.4300	−1.99%	1.4750	−13.59%
Derivative 73	9.0150	13.04%	1.5230	4.39%	1.5320	−10.25%
Derivative 74	9.3700	17.49%	1.3630	−6.58%	1.7120	0.29%
Derivative 75	9.0220	13.13%	1.4800	1.44%	1.6800	−1.58%
Derivative 76	8.4140	5.50%	1.5220	4.32%	1.4980	−12.24%
Derivative 77	9.4620	18.65%	1.3840	−5.14%	1.4730	−13.71%
Derivative 78	8.9870	12.69%	1.6650	14.12%	1.4560	−14.70%
Derivative 79	9.0140	13.03%	1.2830	−12.06%	1.5410	−9.72%
Derivative 80	8.7330	9.50%	1.5340	5.14%	1.5350	−10.08%
Derivative 81	8.5510	7.22%	1.6340	11.99%	1.7030	−0.23%
Derivative 82	8.1340	1.99%	1.5160	3.91%	1.4660	−14.12%
Derivative 83	9.1820	15.13%	1.6460	12.82%	1.4190	−16.87%
Derivative 84	8.4940	6.51%	1.5230	4.39%	1.4390	−15.70%
Derivative 85	8.9160	11.80%	1.5790	8.22%	1.4700	−13.88%
Derivative 86	8.8460	10.92%	1.5280	4.73%	1.4610	−14.41%
Derivative 87	7.9940	0.24%	1.5970	9.46%	1.5430	−9.61%

## Supplementary Materials

The following are available online at https://www.mdpi.com/1660-4601/17/9/3239/s1, Figure S1: Relationship between each QA structure and the associated activities (i.e., IRI, UVI and CI) with (a) marbofloxacin and (b) levofloxacin used as the target molecules, Table S1: Molecular structures of designed QA derivatives.
