# Supplementary material for: Integration of Fuzzy Matter-Element Method and 3D-QSAR Model for Generation of Environmentally Friendly Quinolone Derivatives
Source: Int J Environ Res Public Health. 2020 May 6;17(9):3239. doi: 10.3390/ijerph17093239 (PMC7246649; doi:10.3390/ijerph17093239)
Supplement: Supplementary file 1 [file ijerph-17-03239-s001.pdf]

Supplementary Material

Integration of Fuzzy Matter-Element Method and 3D-QSAR Model for Generation of Environmentally Friendly Quinolone Derivatives

Xixi Li[1], Baiyu Zhang[1*], Wendy Huang[1], Cuirin Cantwell[1] and Bing Chen[1]

Northern Region Persistent Organic Pollution Control (NRPOP) Laboratory, Civil Engineering, Faculty of Engineering and Applied Science, Memorial University, St. John's, NL, Canada, A1B 3X5; emails: xl7724@mun.ca, bzhang@mun.ca, wendyh@mun.ca, cdcantwell@mun.ca, and bchen@mun.ca.

[*] Corresponding author: bzhang@mun.ca

Table S1. Molecular structures of designed QA derivatives

QA derivatives	Molecular structures	QA derivatives	Molecular structures
**QA1**	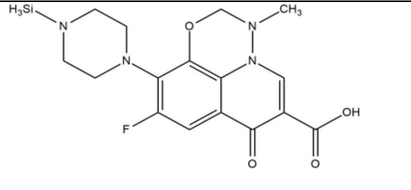	**QA45**	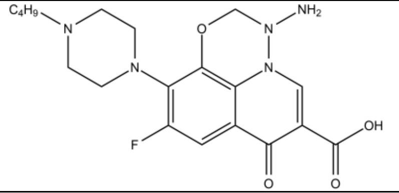
**QA2**	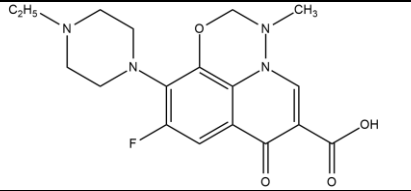	**QA46**	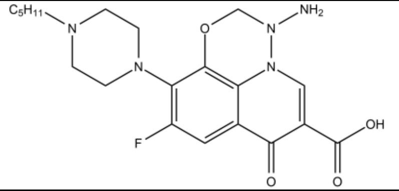
**QA3**	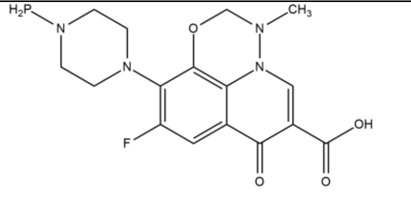	**QA47**	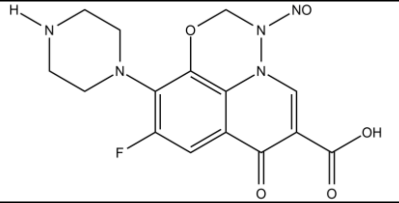
**QA4**	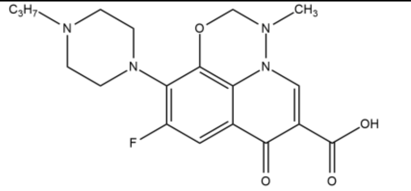	**QA48**	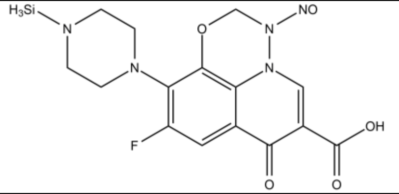
**QA5**	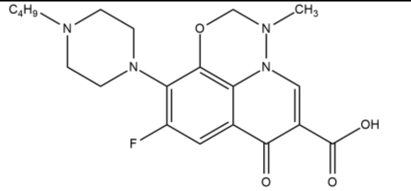	**QA49**	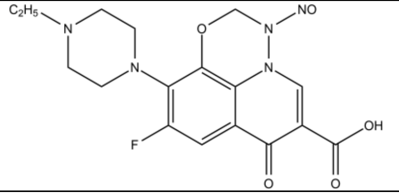
**QA6**	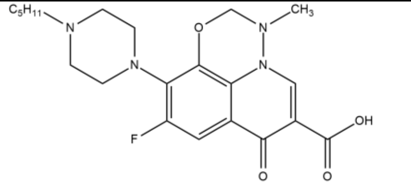	**QA50**	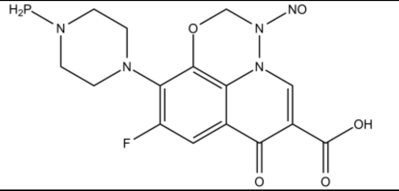
**QA7**	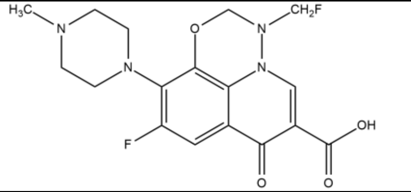	**QA51**	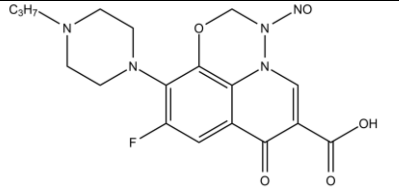
**QA8**	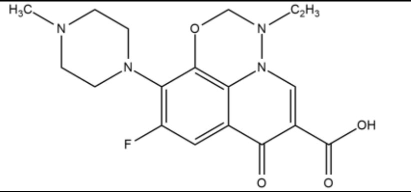	**QA52**	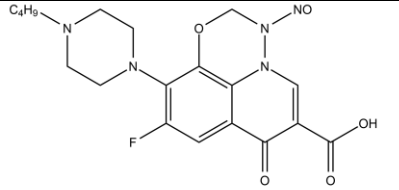

**QA9**	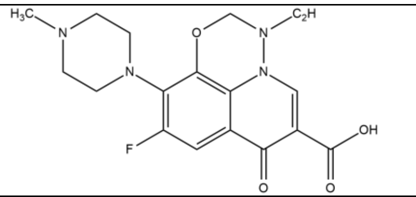	**QA53**	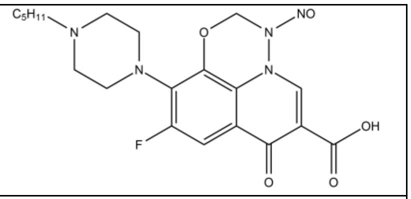
**QA10**	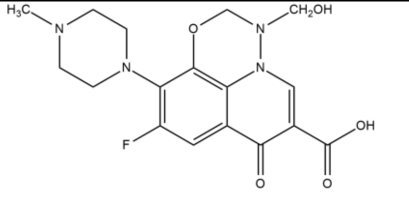	**QA54**	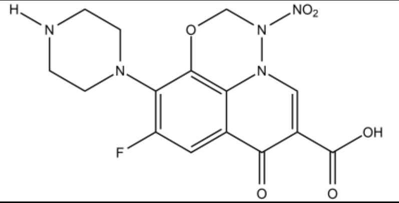
**QA11**	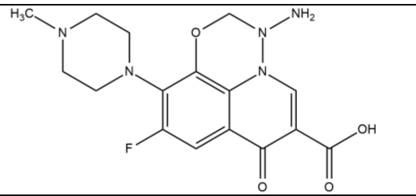	**QA55**	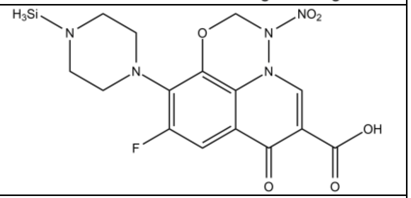
**QA12**	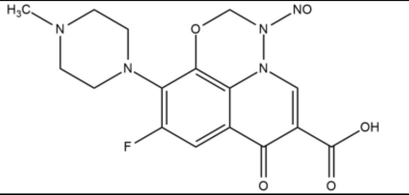	**QA56**	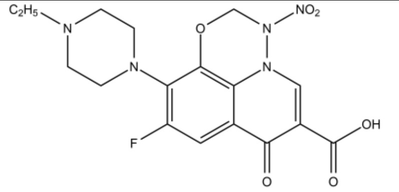
**QA13**	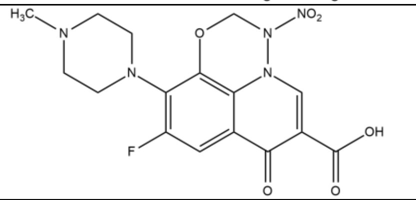	**QA57**	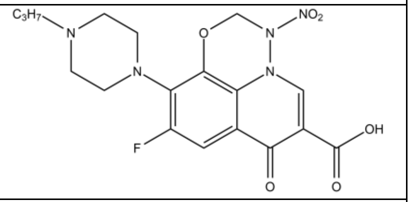
**QA14**	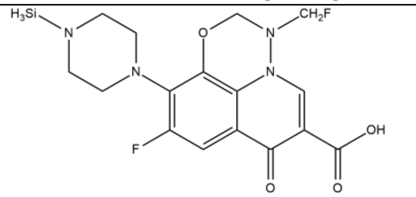	**QA58**	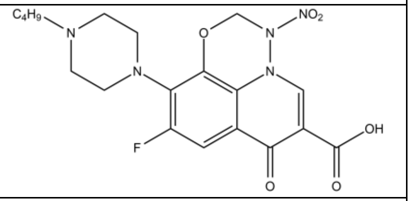
**QA15**	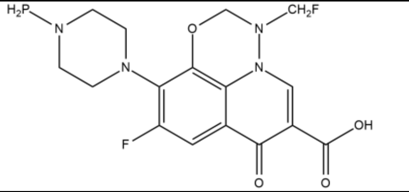	**QA59**	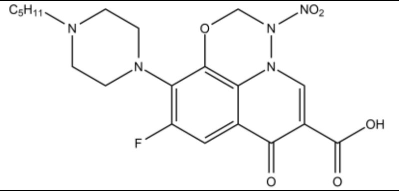
**QA16**	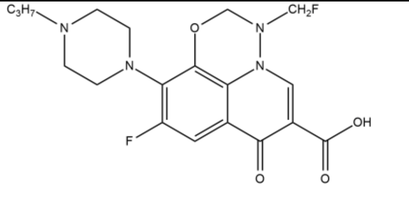	**QA60**	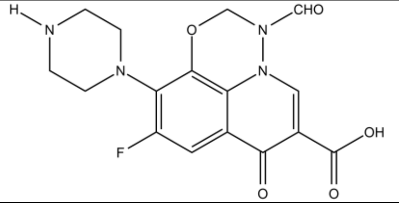

**QA17**	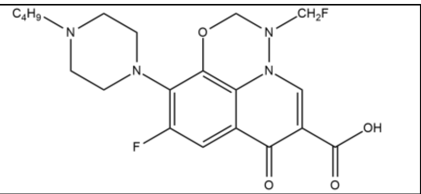	**QA61**	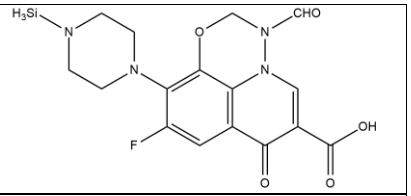
**QA18**	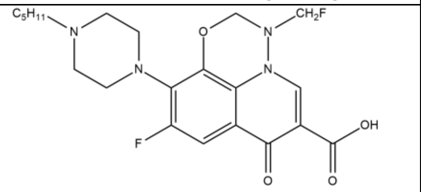	**QA62**	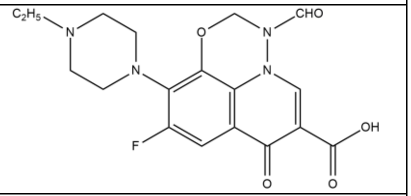
**QA19**	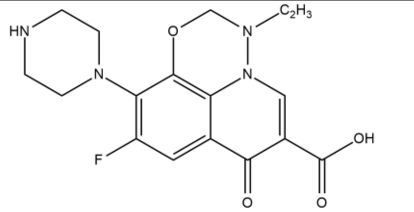	**QA63**	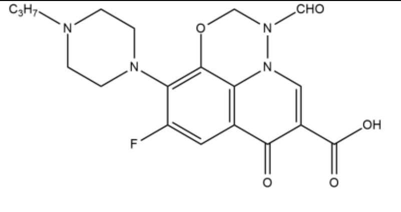
**QA20**	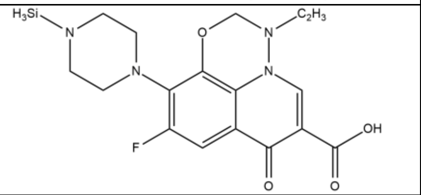	**QA64**	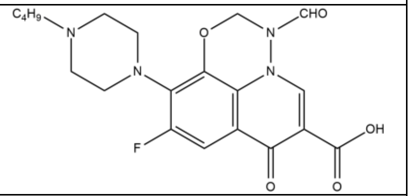
**QA21**	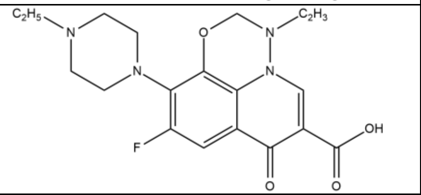	**QA65**	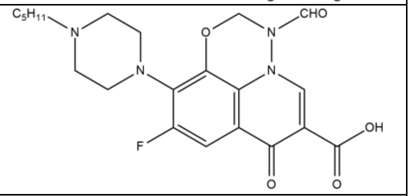
**QA22**	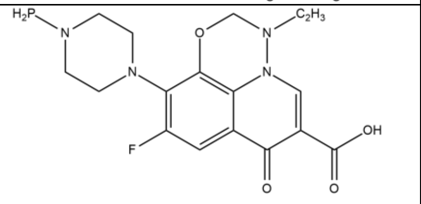	**QA66**	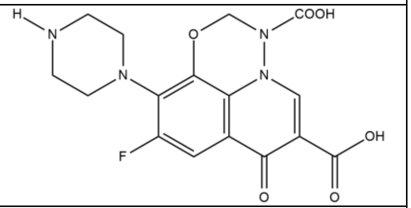
**QA23**	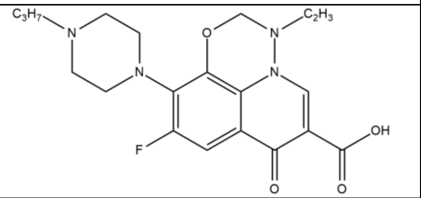	**QA67**	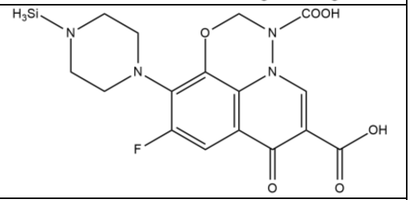
**QA24**	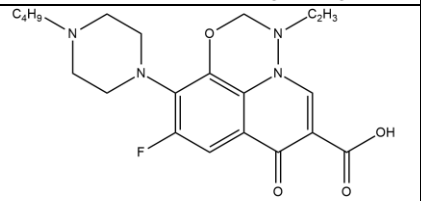	**QA68**	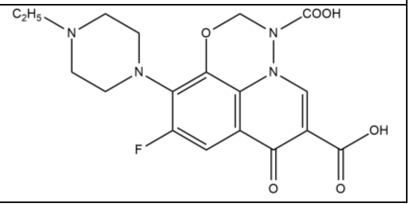

**QA25**	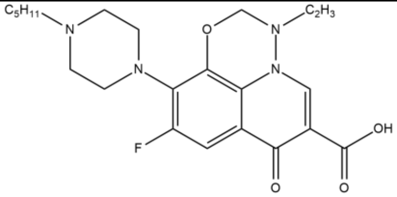	**QA69**	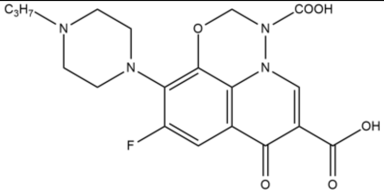
**QA26**	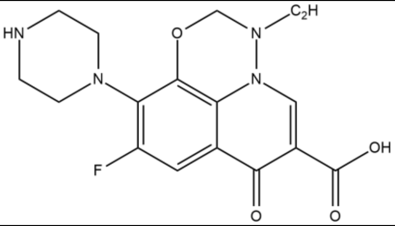	**QA70**	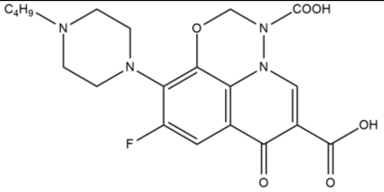
**QA27**	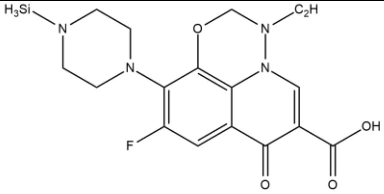	**QA71**	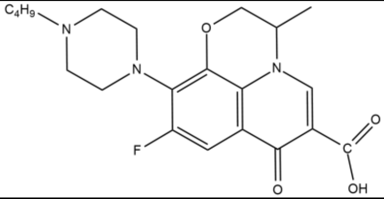
**QA28**	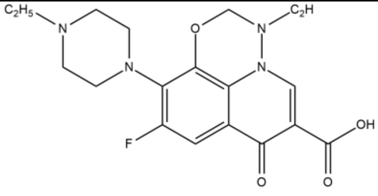	**QA72**	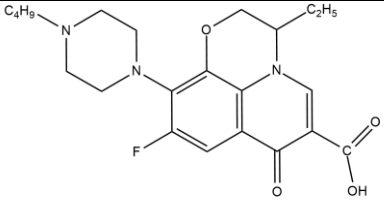
**QA29**	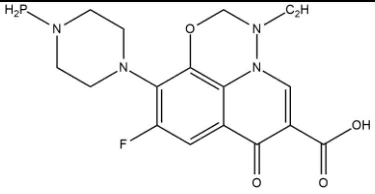	**QA73**	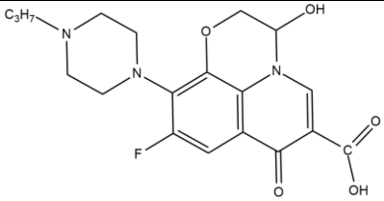
**QA30**	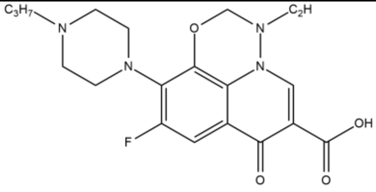	**QA74**	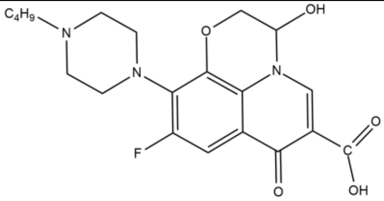
**QA31**	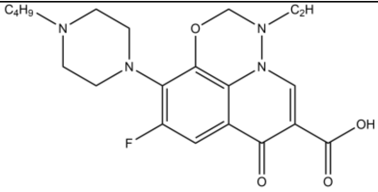	**QA75**	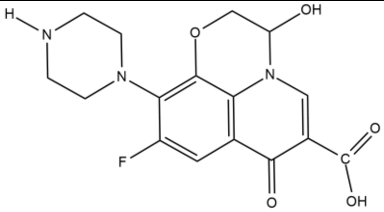
**QA32**	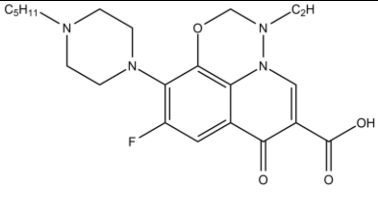	**QA76**	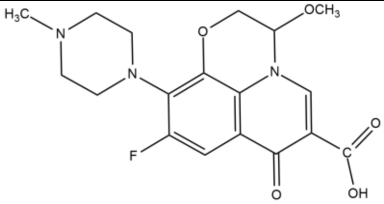

**QA33**	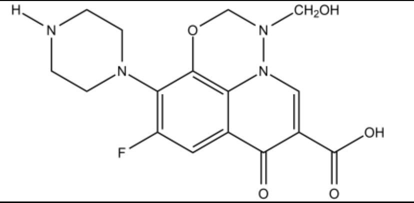	**QA77**	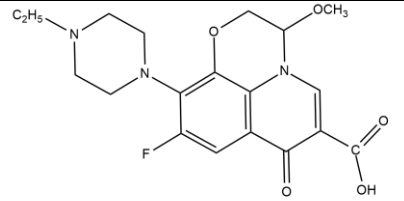
**QA34**	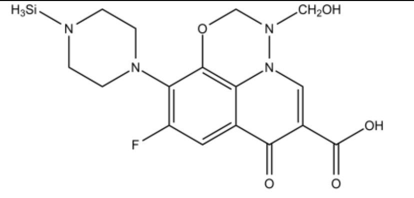	**QA78**	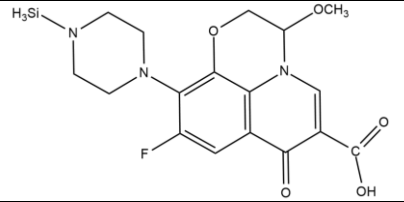
**QA35**	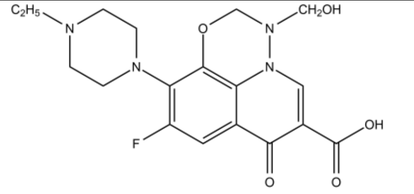	**QA79**	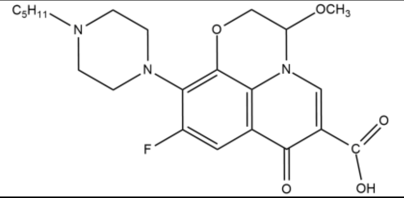
**QA36**	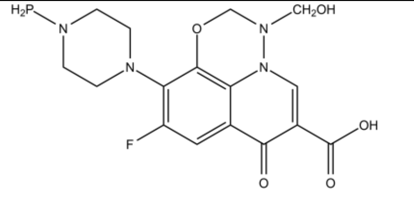	**QA80**	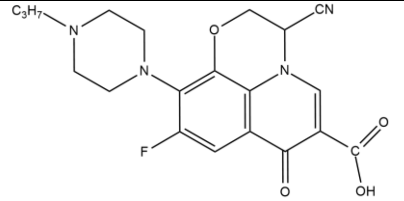
**QA37**	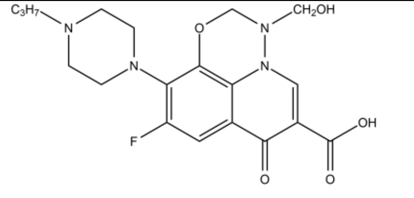	**QA81**	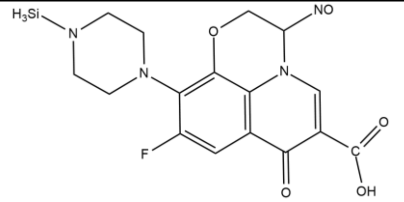
**QA38**	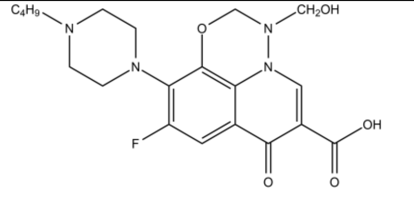	**QA82**	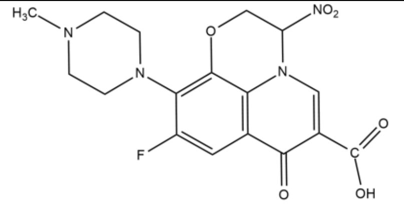
**QA39**	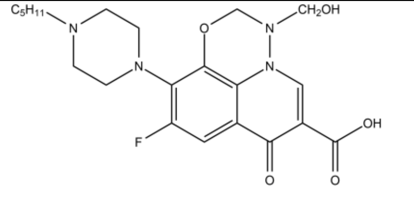	**QA83**	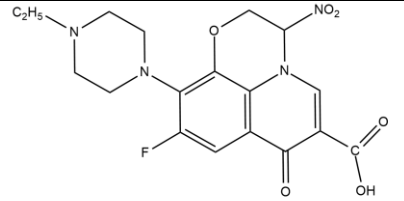
**QA40**	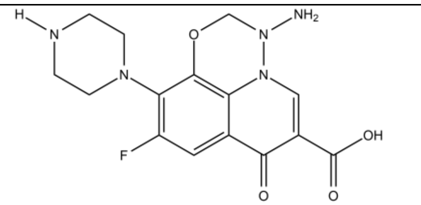	**QA84**	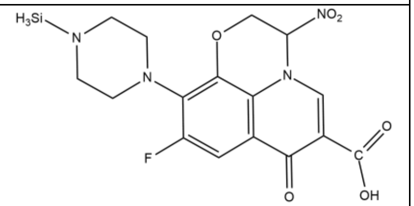

**QA41**	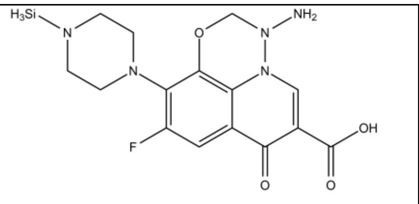	**QA85**	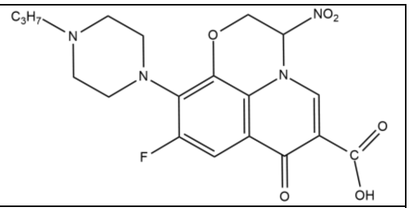
**QA42**	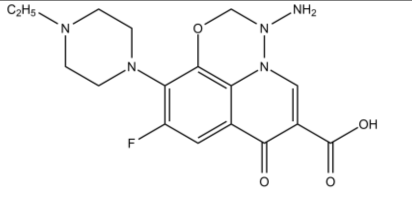	**QA86**	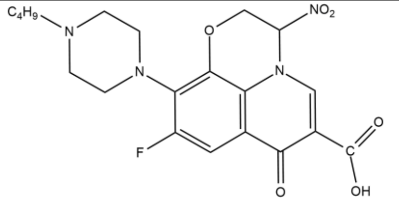
**QA43**	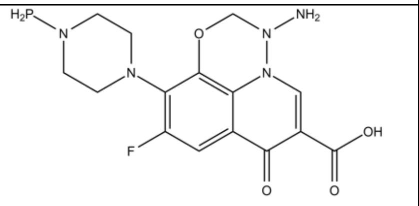	**QA87**	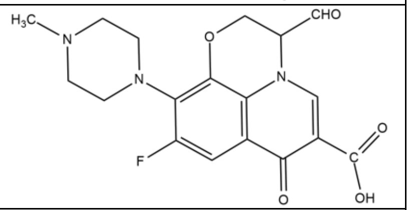
**QA44**	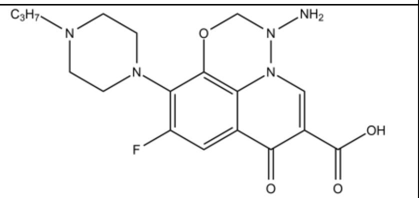		

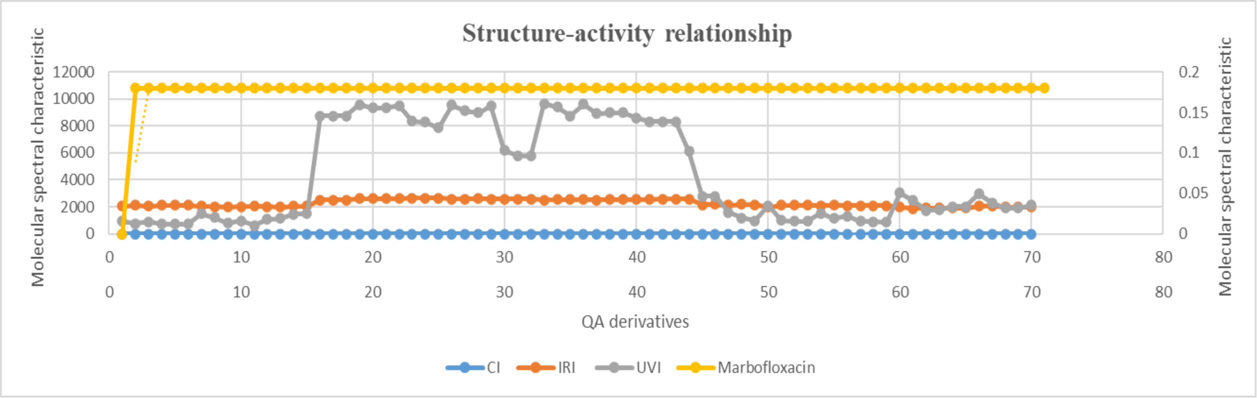

(a)

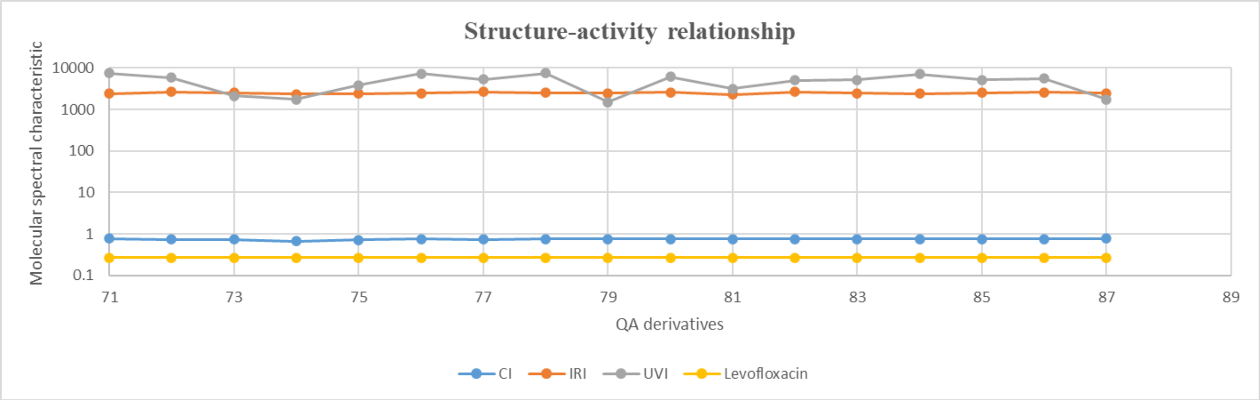

(b)

Figure S1. Relationship between each QA structure and the associated activities (i.e., IRI, UVI and CI) with (a) marbofloxacin and (b) levofloxacin used as the target molecules.
